# Neural Primacy of the Salience Processing System in Schizophrenia

**DOI:** 10.1016/j.neuron.2013.06.027

**Published:** 2013-08-21

**Authors:** Lena Palaniyappan, Molly Simmonite, Thomas P. White, Elizabeth B. Liddle, Peter F. Liddle

**Affiliations:** 1 Centre for Translational Neuroimaging in Mental Health, Division of Psychiatry, University of Nottingham, Nottingham NG7 2TU, UK; 2 Department of Psychosis Studies, Institute of Psychiatry, King’s College London, London SE5 8AF, UK; 3 Nottinghamshire Healthcare NHS Trust, Nottingham NG3 6AA, UK

## Abstract

For effective information processing, two large-scale distributed neural networks appear to be critical: a multimodal executive system anchored on the dorsolateral prefrontal cortex (DLPFC) and a salience system anchored on the anterior insula. Aberrant interaction among distributed networks is a feature of psychiatric disorders such as schizophrenia. We used whole-brain Granger causal modeling using resting fMRI and observed a significant failure of both the feedforward and reciprocal influence between the insula and the DLPFC in schizophrenia. Further, a significant failure of directed influence from bilateral visual cortices to the insula was also seen in patients. These findings provide compelling evidence for a breakdown of the salience-execution loop in the clinical expression of psychosis. In addition, this offers a parsimonious explanation for the often-observed “frontal inefficiency,” the failure to recruit prefrontal system when salient or novel information becomes available in patients with schizophrenia.

## Highlights

• A salience-executive loop emerges on fMRI whole-brain Granger causal analysis
• At rest, DLPFC has inhibitory Granger influence on the salience network
• In schizophrenia, the salience-executive interaction is diminished
• Visual cortex fails to influence the salience network in schizophrenia

## Teaser

Palaniyappan et al. show that in patients with schizophrenia, a reciprocal salience-execution loop involving anterior insula and dorsolateral prefrontal cortex disintegrates along with a failure of hierarchical influence from sensory regions to the salience processing system.

## Introduction

Several functional brain imaging studies support the existence of two “task-positive” brain systems that facilitate efficient performance of tasks that require focused attention (Seeley et al., 2007). One of these large-scale networks, termed the salience network (SN), is anchored in the right anterior insula (rAI) and dorsal ACC (dACC) and has predominant limbic and subcortical components. The SN is involved in integrating external stimuli with internal homeostatic context, thus marking objects that require further processing (Menon and Uddin, 2010; Seth et al., 2011; Singer et al., 2009). A second network comprised of the dorsolateral prefrontal cortex (DLPFC) and lateral parietal regions, termed the central executive network (CEN), operates on the identified salient stimuli to enable task performance (Seeley et al., 2007). These two networks are thought to interact at various levels to enable coordinated neural activity (Medford and Critchley, 2010). First, the rAI is thought to causally influence the anticorrelation between the CEN and a set of brain regions involved in self-referential activities that constitute the default mode network (DMN) (Sridharan et al., 2008). Thus, the rAI has a strong causal influence enabling the recruitment of contextually relevant brain regions. Second, along with dACC and thalamus, rAI forms a tonic-alertness loop that forms a vital subcortical-limbic system in a hierarchical attention-processing stream (Sadaghiani et al., 2010). In addition, during task performance, the dACC acts in conjunction with the DLPFC to form a cognitive control loop that modulates the behavioral response (Miller and Cohen, 2001).

Converging evidence from structural and functional neuroimaging studies indicate a crucial role for both the rAI (Palaniyappan and Liddle, 2012) and the DLPFC (Callicott et al., 2000; Weinberger et al., 1992) in the pathophysiology of schizophrenia. A number of neuropathological and imaging studies have found abnormalities in the DLPFC, with robust evidence implicating a failure of excitatory-inhibitory neuronal balance in this region (Lewis et al., 2005). Several pooled analyses of structural imaging studies have confirmed that the most consistent gray matter abnormalities across the different stages of schizophrenia occur in the nodes of the SN, especially the anterior insula (Ellison-Wright et al., 2008; Glahn et al., 2008). fMRI studies suggest that an inefficient recruitment of the frontoparietal executive system is often noted alongside SN dysfunction during task performance (Hasenkamp et al., 2011; Kasparek et al., 2013; Minzenberg et al., 2009; Nygård et al., 2012). The presence of SN dysfunction in schizophrenia has also been shown in studies seeking instantaneous functional correlations (also known as functional connectivity) in the blood oxygen level-dependent (BOLD) time series between the rAI and several nodes of the SN (Guller et al., 2012; Pu et al., 2012; Tu et al., 2012), and this within-network SN dysconnectivity is related to cognitive dysfunction (Tu et al., 2012). Similar findings of reduced connectivity within the SN in schizophrenia also emerge when seeking time-lagged (−5 to +5 s) rather than instantaneous correlations between the BOLD signals from brain regions constituting large-scale networks (White et al., 2010). It is possible that the disintegration of the salience processing system anchored on the rAI has a causal role in the inefficient cerebral recruitment noted in schizophrenia. To our knowledge, no neuroimaging studies have so far investigated whether a failure in the feedforward causal influence from the salience processing system to the executive system is present in schizophrenia.

Following the terminology of Friston (1994) in this Article, we employ the term functional connectivity (FC) to denote the instantaneous, zero-time lagged correlation between brain activity occurring at spatially distinct sites. Correlation does not demonstrate a causal relationship between variables and therefore the existence of functional connectivity does not imply that activity in one region causes that in another or even that the regions have a direct neural connection. Brain regions showing significant FC are functionally coupled and may reflect components of a single but spatially distributed system (i.e., a large-scale brain network). Granger causal connectivity is a measure of effective connectivity; the presence of Granger causal connectivity from a region A to another region B implies that the neuronal activity in region A precedes and predicts the neuronal activity that occurs in region B. These two regions, A and B, may not show instantaneous functional coupling that is characteristic of a single large-scale system. Thus, Granger causal analysis (GCA) is a more useful approach to study the causal relationships that may exist across networks.

To investigate the “causal” influences between the salience processing and the executive systems, we employed Granger causality analysis in task-free resting-state fMRI. Task-free conditions minimize potentially confounding effects of between-group performance differences and permit the investigation of fundamental components of neurophysiological function. We hypothesized that the “causal” influence of the rAI over the multimodal brain regions constituting the executive system will be reduced in schizophrenia. We also predicted that any abnormality in the feedforward influence would be accompanied by a reciprocal diminution of the feedback from the executive system to the rAI, resulting in a dysfunctional salience-execution loop in patients. In addition, using a mediation model, we studied the relationship between the abnormalities in the functional connectivity of the SN and the “causal” outflow from the salience processing to the executive system. Finally, we investigated whether the illness severity in patients is predicted by the dysfunction of the salience-execution loop.

## Results

### Demographic and Clinical Variables

The demographic and clinical characteristics of the sample are presented in Table S1 available online. Patients did not differ from the controls in terms of age (mean (SD) age in patients = 34.5(9.1), controls = 33.5(9.1), *t*(71) = 0.46, p = 0.65), gender (females/males = −10/25 in controls; 9/29 in patients, chi-square p = 0.63), handedness (right/left = 33/5 in patients; 31/4 in controls, chi-square p = 0.82), and parental Socioeconomic Scale (SES) score (mean (SD) in patients = 2.4(1.5), controls = 2.1(1.3), *t*(71) = 0.79, p = 0.43). Patients had a mean current symptom burden of 12.1 units (SD = 7.3; range 1 to 25) measured using the Symptoms and Signs in Psychotic Illness (SSPI) (out of a maximum possible score of 80).

### Granger Causality Analysis

**Figure 1:**
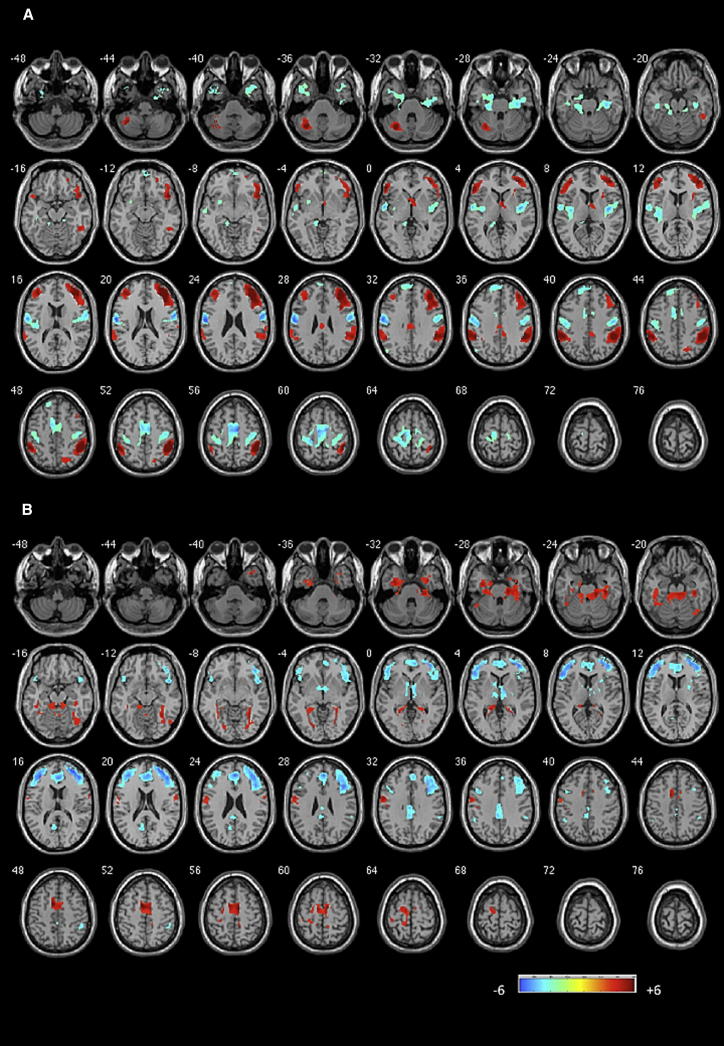
Granger Causal Influence to and from the Right Anterior Insula (A) The influence of right anterior insula on the rest of the brain (x to y). (B) The influence of regions from the rest of the brain on right anterior insula (y to x). The figures show the results of the one-sample t test of GCA maps on all subjects (patients and controls). Illustrations drawn on a single subject structural image showing axial slices using xjview at p < 0.001 uncorrected, k = 30. Color bar shows a scale of T values. Warm colors suggest excitatory influence, while cold colors suggest inhibitory influence. See also Tables S2, S5, and S6 and Figures S4 and S5.

In the entire sample (patients and controls, one-sample t test), rAI exerted a significant excitatory influence on the bilateral DLPFC, inferior parietal regions, and left cerebellar crus. Significant inhibitory influence of the rAI was noted on bilateral supplementary motor regions and bilateral precentral regions, in addition to right posterior insula. Bilateral DLPFC in turn had a significant inhibitory influence on the rAI. In addition, dACC and posterior cingulate cortex (PCC) had significant inhibitory influence, while preSMA and temporal pole had significant excitatory influence on the rAI. These results are shown in Figure 1 and Table S2.

**Figure 2:**
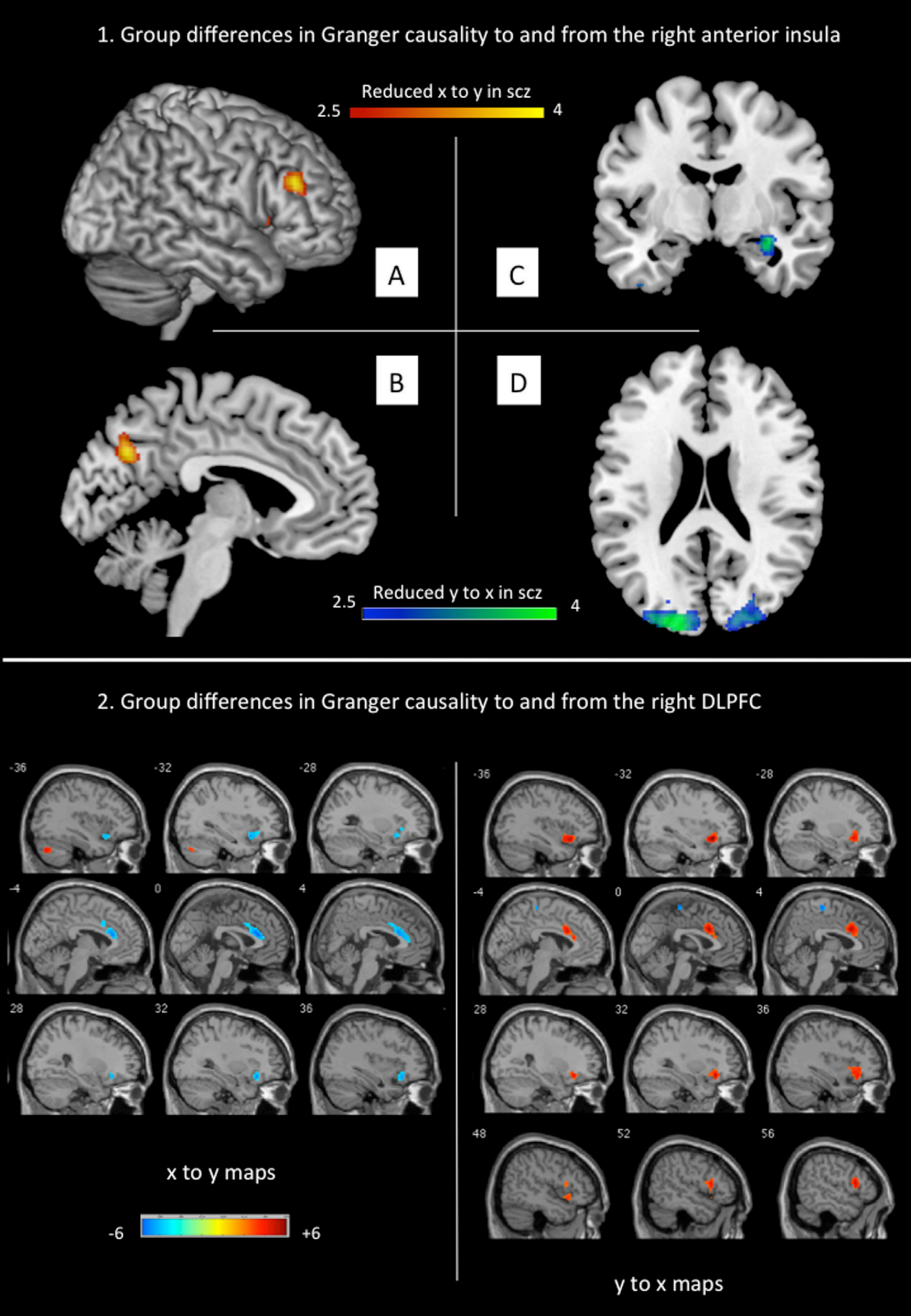
Group Differences in Granger Causal Influences Top: (1) group differences in Granger causality to (y to x) and from (x to y) the right anterior insula in patients with schizophrenia compared to healthy controls. Illustrations drawn on a single subject structural image with slices selected for the best display of regions showing differences in the two-sample t test. Color bar shows a scale of T values. Blue-green areas show regions where patients had reduced y to x path coefficients (i.e., less excitatory influence on the insula) than controls, while red-yellow-colored areas show regions where patients had reduced x-to-y (i.e., less excitatory influence from the insula) path coefficients than controls. (A) Surface-rendered image showing right DLPFC region with most significant reduction in the Granger causal influence from the right anterior insula. (B) Precuneus (x = −4). (C) Hippocampal formation (y = −6). (D) Bilateral visual cortex (z = 18). Bottom: (2) group differences in Granger causality to (y to x) and from (x to y) the right DLPFC in patients with schizophrenia compared to healthy controls. Illustrations drawn on a single subject structural with slices selected for the best display of regions showing differences in the two-sample t test. Left: group differences in x-to-y maps; right: group differences in y-to-x maps. Color bar shows a scale of T values. Blue areas show regions where patients had greater (i.e., less inhibitory) path coefficients than controls, while red-colored areas show regions where controls had greater (i.e., more excitatory) path coefficients than patients. See also Table S7 and Figure S3.

Two-sample t tests revealed significant differences between patients and controls in the “causal” outflow from the rAI to the rDLPFC. In controls, the rAI exerted a significant excitatory influence on right DLPFC (t(34) = 7.42, corrected p < 0.001), while in the patients, this influence was weak (t(37) = 2.06, uncorrected p = 0.047). In addition, there was a group difference in the effect of rAI on precuneus at an uncorrected threshold (p < 0.001, k = 30), where the controls exhibited an excitatory influence (t(34) = 3.14, uncorrected p = 0.004), while the patients exhibited an inhibitory influence (t(37) = −2.18, uncorrected p = 0.036). Patients also showed a significant reduction in the “causal” influence from bilateral visual cortex and right hippocampal formation to the insula when compared to controls. These group differences are shown in Figure 2 and Table 1.

**Figure 3:**
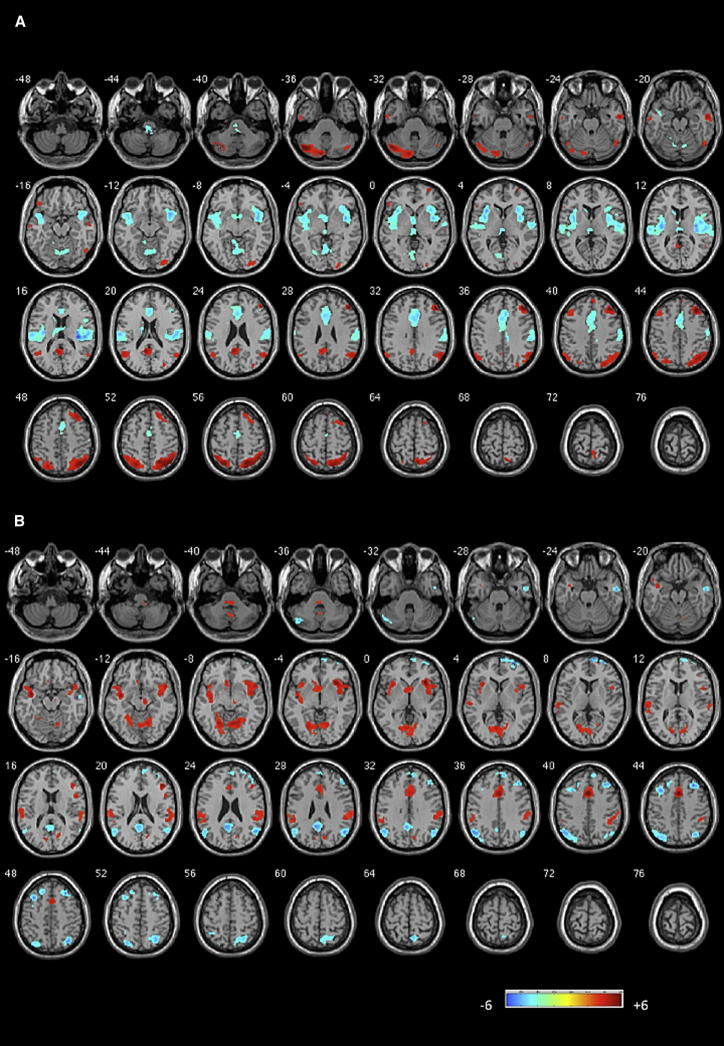
Granger Causal Influence to and from the Right Dorsolateral Prefrontal Cortex (A) The influence of right dorsolateral prefrontal cortex (rDLPFC) on the rest of the brain (x to y). (B) The influence of rest of the brain on rDLPFC (y to x). The figures show the results of the one-sample t test of GCA maps on all subjects (patients and controls). Illustrations drawn on a single subject structural image showing axial slices using xjview at p < 0.001 uncorrected, k = 30. Color bar shows a scale of T values. Warm colors suggest excitatory influence, while cold colors suggest inhibitory influence. See also Table S3.

In order to investigate the effects of influences of the rDLPFC on the rest of the brain, we performed voxelwise GCA using a 6 mm spherical region of interest (ROI) placed in the rDLPFC node showing the significant group difference. The SN was the primary site of dysfunctional “causal” influence on the rDLPFC in patients. Patients had a significantly reduced excitatory effect from the bilateral (more ventral) insula and the dACC to the rDLPFC in addition to a significant loss of inhibitory effect of the rDLPFC on the bilateral anterior insula and dorsal ACC (Figure 2; Table 2). The results of the one-sample t tests of GCA based on the rDLPFC seed are presented in Figure 3 and Table S3.

None of the x-to-y or y-to-x path coefficients from the rAI or the DLPFC seed regions showed significant correlations with antipsychotic dose equivalents (all p > 0.2). The GCA analysis using a homologous left anterior insula seed revealed that the salience-execution loop disturbances are predominantly right lateralized in schizophrenia (further details are presented in the Supplemental Information and in Figures S4 and S5 and Tables S5 and S6).

### Relationship with Illness Severity

To relate the illness severity to GCA coefficients in patients, we conducted three principal component analyses to extract an illness severity factor, a factor representing the integrity of “causal” interactions within the salience-execution loop (rAI, rDLPFC, and dACC), and a factor representing visual inflow to rAI. A multiple regression analysis was then conducted as described in the Experimental Procedures section. The model had a significant fit (F[3,34] = 4.03, R^2^ = 0.26, p = 0.015). Illness severity was significantly predicted by both reduced integrity of the salience-execution loop (β = −0.71; t = −2.8, p = 0.008) and reduced integrity of the visual inflow to the rAI (β = −0.32; t = −2.1, p = 0.04). Antipsychotic dose had a trend-level association with higher dose being prescribed for patients with more severe illness (β = 0.27; t = 1.9, p = 0.064). Further details are presented in the supplemental material (Table S7 and Figure S3).

### Functional Connectivity

**Figure 4:**
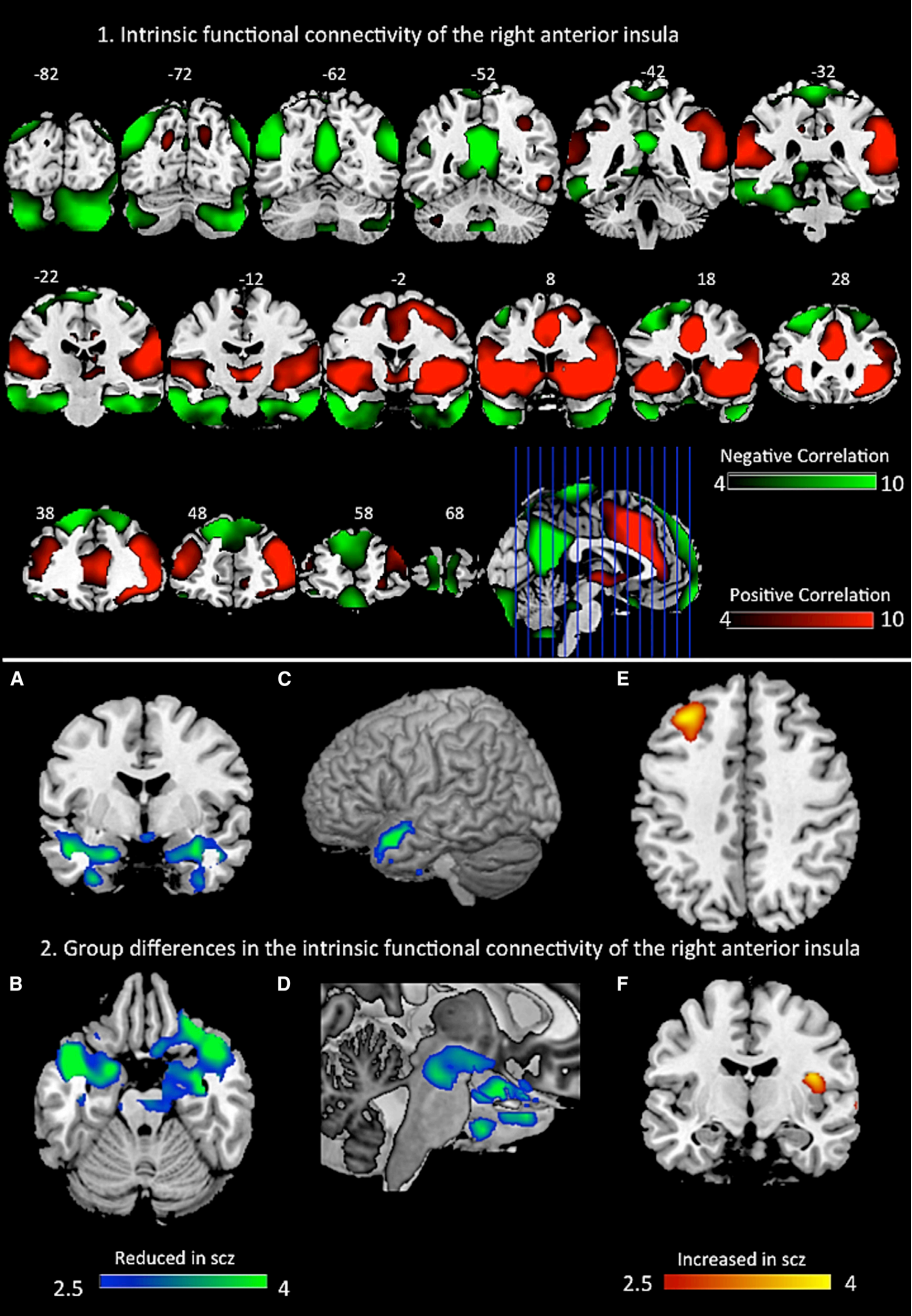
Functional Connectivity of the Right Anterior Insula Top: (1) functional connectivity of the right anterior insula. The figure depicts the results of the one-sample t test of z-transformed voxelwise correlation maps on all subjects (patients and controls). Illustrations drawn on a single subject structural image showing coronal slices using MRICron at familywise error-corrected p < 0.05, k = 30. Color bar shows a scale of T values. Red-colored areas show positive correlation, while green-colored areas show anticorrelation with the seed region. Bottom: (2) group differences in functional connectivity of the right anterior insula in patients with schizophrenia compared to healthy controls. Illustrations drawn on a single subject structure with slices selected for the best display of regions showing differences in the two-sample t test. Color bar shows a scale of T values. Red-yellow-colored areas show regions where patients had greater Fisher’s r-to-z correlation scores than controls, while green-blue-colored areas show regions where patients had lower z Fisher’s r-to-z scores than controls. (A and B) Amygdala/parahippocampal region (y = −6, z = −21). (C) Surface-rendered image showing left superior temporal pole. (D) Magnified cut section (x = 6) of surface-rendered image showing periaqueductal gray matter. (E) Left middle frontal region (z = 42). (F) Right posterior insula (y = −12). See also Table S4 and Figure S2.

One-sample t tests of FC maps reflecting functional coupling between rAI and the rest of the brain revealed significant positive correlation with several regions constituting the SN (bilateral anterior insula, extending to anterior and midcingulate, bilateral inferior frontal, middle frontal and superior temporal gyrus, supramarginal gyrus, putamen, and thalamus). In addition, positive correlation was also noted at right middle temporal gyrus and small clusters located bilaterally in the dorsal precuneus. Extensive anticorrelation was noted between the rAI seed and nodes constituting the DMN including the PCC/ventral precuneus, angular gyrus, and parahippocampal region. The results are shown in Figure 4 and Table S4.

Two-sample t tests comparing the FC maps of patients and controls revealed significant differences in the rAI connectivity with key paralimbic regions including bilateral temporal pole, parahippocampal region, and the amygdala. In the right temporal pole, patients showed no significant functional connectivity (one-sample t(37) = 0.24, p = 0.81), while controls showed a significant positive correlation (one-sample t(34) = 7.42, corrected p < 0.001). At the left temporal pole, patients showed an anticorrelation (one-sample t(37) = −4.9, corrected p < 0.001), while controls had a positive correlation (one-sample t(34) = 3.78, corrected p < 0.001).

A similar dissociation in the FC between the two groups was also noted in other limbic clusters when using an uncorrected threshold of p < 0.001, k = 30 (periaqueductal gray matter [two-sample (t) = 3.74, k = 60; patients, one-sample t(37) = −3.06, p = 0.004; controls, one-sample t(34) = 2.42, p = 0.021] and right parahippocampal/amygdala [two-sample (t) = 4.36, k = 159; patients, one-sample t(37) = −2.72, p = 0.010; controls, one-sample t(34) = 3.51, p = 0.001]). Left DLPFC and left posterior insula showed significant group difference (schizophrenia > controls) at the uncorrected threshold. At the left DLPFC, a significant anticorrelation in controls (one-sample t(34) = −5.88, p < 0.001) and absence of significant correlation in patients (one-sample t(37) = 0.41, p = 0.69) was noted. At the left posterior insula, a significant positive correlation was seen in the patients (one-sample t(37) = 5.75, p < 0.001), while controls had no significant correlation (one-sample t(34) = 0.70, p = 0.49). The group differences are shown in Table 3 and Figure 4.

The eigenvariate derived from the clusters showing either reduced or increased FC in patients showed no significant correlations with antipsychotic dose equivalents (both p > 0.2).

### Mediation Analysis

A mediation analysis (see Experimental Procedures) was conducted to study the effect of aberrant rAI FC (“rAI-temporolimbic dysconnectivity”) on the diagnostic difference in the GCA coefficient from rAI to rDLPFC. The diagnostic difference in the rAI to the rDLPFC outflow was significantly mediated by the reduced within-network connectivity in the SN. The mediation model had a significant fit (R^2^ = 0.18;F[1,71] = 16.1, p = 0.0001; total effect coefficient = 0.076). The diagnosis of schizophrenia had a significant direct effect on the influence from the insula to the DLPFC (direct coefficient (SD) = 0.05 (0.19), p = 0.02). The coefficient representing indirect effect, due to the rAI-temporolimbic dysconnectivity was 0.02 (SD = 0.09), 95% confidence limits from bootstrap test (0.045–0.003, number of simulations = 5,000). Thirty percent of the total effect of the diagnosis on the rAI-DLPFC interaction was explained by the temporolimbic dysconnectivity. The mediation model tested in the current study is illustrated in Figure S2.

## Discussion

### Granger Causality Analysis

Though deficits in brain regions involved in processing stimulus salience and cognitive control have been repeatedly shown in schizophrenia, to our knowledge this is the first study that directly investigates the “causal” relationship between the dysfunctions observed in these two systems. Using Granger causal analysis, we infer that patients with schizophrenia have significantly reduced neural influence from the rAI, a key node in the salience processing system, to the DLPFC, a crucial node in the executive loop. Further, the most significant abnormality in the influences to and from the DLPFC in patients with schizophrenia involved the nodes of the SN—the dACC and the anterior insula. These observations confirm our primary hypothesis that the interaction between the paralimbic salience processing system and the multimodal executive system is significantly diminished in schizophrenia (Figure S1).

Van Snellenberg et al. (2006) concluded that the magnitude of working memory performance reduction in schizophrenia is associated with degree of attenuation of DLPFC activation. Inefficient DLPFC recruitment is apparent when the task becomes more challenging (Potkin et al., 2009). It is not simply the failure to recruit frontoparietal systems that is associated with the reduced task performance, but there is a conjoint failure to deactivate or “switch-off” the task-irrelevant DMN system that includes multimodal midline structures such as the ventromedial prefrontal cortex (Nygård et al., 2012) and PCC/precuneus (Hasenkamp et al., 2011), in addition to parahippocampal regions (Whitfield-Gabrieli and Ford, 2012). Successful anticorrelation between these two networks appears crucial for effective task performance, and this anticorrelation is affected in schizophrenia (Whitfield-Gabrieli and Ford, 2012). The SN has been proposed to regulate the two competing brain systems (Seeley et al., 2007; Sridharan et al., 2008). Our observation that, during rest, the influence of the rAI on the DLPFC and to some extent on the precuneus is diminished in schizophrenia suggests that the inefficient cerebral recruitment associated with cognitive dysfunction in schizophrenia is likely to result from a failure of paralimbic-multimodal integration rather than a focal DLPFC dysfunction alone. Further, the abnormal reciprocal influence from DLPFC was more ventrally located in the insula, highlighting the somewhat selective loss of prefrontal influence predominantly directed to the socioemotional frontoinsular cortex (Kurth et al., 2010).

In patients with schizophrenia, both the excitatory influence of dACC onto DLPFC and the inhibitory influence from the DLPFC onto dACC were significantly reduced. ACC is frequently coactivated with DLPFC during task performances, irrespective of the nature of the stimulus and response (Koski and Paus, 2000). Several computational models suggesting bidirectional flow of information between ACC and DLPFC have been put forward, with both feedforward and feedback influences proposed in addition to indirect influences via other brain structures (Mars et al., 2012). But to date, the detailed topography of these circuits remains unclear. Tracer injection studies from rhesus monkeys indicate that ACC exerts both prominent excitatory and inhibitory effects on the DLPFC (Medalla and Barbas, 2009). Barbas (2000) suggests that DLPFC has no direct limbic connections, though it is likely to access limbic signals via paralimbic structures including the ACC. Interestingly, in schizophrenia, at least in the superficial layers of the ACC, inhibitory neurons appear to be reduced in their density (Reynolds et al., 2001). The prominent failure of the bidirectional communication between the dACC and the DLPFC observed in our sample suggest that the transfer of limbic signals onto the DLPFC is abnormal in schizophrenia. It is, however, important to note that both ACC and DLPFC are large brain regions with significant heterogeneity in the functional specialization of neuronal subsets (Johnston et al., 2007); hence generalizing the present results derived from selected coordinates to the entire dACC/DLPFC circuitry may not be appropriate.

It is worth noting that in the original description of the SN using FC, Seeley et al. (2007) hypothesized that in task-free settings, the SN and CEN are negatively correlated with the DMN but are minimally correlated with one another. Our observations suggest that in fact, at rest, while the SN exerts an excitatory influence on the DLPFC, in turn the DLPFC exerts an inhibitory influence on the SN. It is possible that a well-balanced salience-execution loop exists during rest, and on the arrival of appropriate stimulus that violates expectancies of the resting state, this balance is perturbed with an increase in the positive influence of SN over the DLPFC and a reduction in the negative influence of the DLPFC over the SN leading to a reverberating excitatory process in this loop. This speculation requires verification from direct electrophysiological studies during task performance.

Patients with reduced “causal” influence within the salience-execution loop had poor occupational and sociofunctional ability, cognitive dysfunction characterized by reduced processing speed, and higher symptom burden in the domains of disorganization, psychomotor poverty, and reality distortion despite antipsychotic treatment. A similar, albeit less prominent, relationship was observed between reduced visual inflow to rAI and higher illness severity in patients. This predictive relationship observed between the impairments in the directed influences within the salience-execution loop and the symptom burden validates the notion that an impaired “switching” function of the SN contributes to several core symptoms of schizophrenia and contributes to functional disability (Palaniyappan et al., 2012). Given that the patients in this sample were in a clinically stable phase, this relationship is likely to reflect the role of the salience processing system on the “trait-like” aspects of the clinical presentation of schizophrenia. In the present study, both reduced visual inflow to the rAI and the impaired “causal” connectivity within the salience-execution loop predicted reduced processing speed. This reconciles previous findings that reported impaired processing speed both in relation to functional hypofrontality (Molina et al., 2009) and structural dysconnectivity involving occipitofrontal fasciculi (Palaniyappan et al., 2013) and affirms the cardinal role of rAI in the pathophysiology of schizophrenia.

We did not predict a reduction in the “causal” inflow from the visual cortex to the rAI in schizophrenia a priori. Nevertheless, in line with the mounting evidence implicating a failure of bottom-up processes in psychosis (Javitt, 2009) and their relationship with anhedonia, apathy, negative symptoms, and cognitive dysfunction (Javitt, 2009), our results suggest that insular dysfunction is characterized by both a reduced visual inflow and frontal outflow. Thus, the SN is likely to form a crucial link in the hierarchical processing (sensory regions → salience network → executive network) abnormalities that contribute to the clinical expression of schizophrenia.

### Functional Connectivity

We observed a prominent loss of instantaneous positive correlation between the rAI and bilateral temporal pole. Unlike healthy controls who showed a positive correlation, patients showed an anticorrelation between rAI and bilateral medial temporal lobe structures. Temporal pole is a prominent paralimbic region with a crucial role in socioemotional processing (Olson et al., 2007). In patients with schizophrenia, medial temporal structures form a significant component of the task-negative DMN (Garrity et al., 2007) but often fail to “switch-off” during cognitive tasks. The presence of significant disconnectivity between rAI and temporolimbic system suggests that the abnormalities in the SN-mediated switch-off of DMN during task performance could affect the medial temporal region in particular. Further, temporal poles have a role in feeding semantically processed environmental stimuli to the insula (Craig, 2009). The temporoinsular disconnectivity in schizophrenia merits further investigation in this context.

Meyer-Lindenberg et al. (2005) observed that the attenuated deactivation of the temporolimbic system is related to frontal inefficiency in schizophrenia. We find that the degree of rAI-temporolimbic functional dysconnectivity in schizophrenia explains a significant portion of the reduced influence of insula on DLPFC, suggesting that an adaptive paralimbic gating of executive system is disorganized in patients (Dichter et al., 2010).

### Clinical Relevance and Future Directions

Plasticity of functional networks is now well recognized (Lewis et al., 2009), though the brain network that requires targeting in order to reverse a cognitive or behavioral deficit continues to be speculative. By demonstrating the central role of insular dysfunction in the disrupted salience processing and executive systems in schizophrenia, the present study specifies that SN reorganization could be a treatment target in schizophrenia. Several interesting therapeutic opportunities have emerged in recent times indicating the feasibility of modulating the function of the SN.

The emergence of repetitive transcranial magnetic (rTMS) and direct current stimulation (tDCS) approaches offer very promising noninvasive physical interventions to modulate network plasticity. Meta-analysis indicates that rTMS applied to temporoparietal junction ameliorates persistent hallucinations in schizophrenia (Slotema et al., 2012), with preliminary evidence suggesting that modulation of the anterior insular connectivity predicts treatment response (Vercammen et al., 2010). Anterior insula, due to its sequestrated location, is often considered to be beyond the reach of rTMS or tDCS approaches. Our current observation of the existence of an rAI-rDLPFC “causal” feedback loop raises the possibility of modulating anterior insula, by focused targeting of the more accessible rDLPFC. In addition to neurostimulation approaches, certain cognitive approaches also appear to exert a specific influence on the SN. One cognitive approach with several features suggestive of regulating the function of the SN is mindfulness training (Zeidan et al., 2011). Another potential approach recently shown to manipulate the interaction between the SN and other distributed networks in schizophrenia is neurofeedback using real-time fMRI (RtfMRI) or electroencephalogram (Ruiz et al., 2013).

Eventually, an optimum combination of pharmacological manipulation to improve plasticity of brain networks, along with targeted cognitive training/neurostimulation to influence network reorganization, is likely to provide the most robust approach to address dysfunctional SN in schizophrenia. Though there are limited data demonstrating pharmacological modulation of large-scale brain networks, early evidence implicates dopamine in the interaction of the SN with subcortical sites (Cole et al., 2013) and GABA/Glutamate in the within-network connectivity of the SN and the interaction of the SN with other large-scale networks (Forget et al., 2010; Palaniyappan et al., 2012).

### Strengths and Limitations

We employed a whole-brain Granger causality analysis, instead of choosing a priori ROIs, which enabled us to study the Granger causal influence of the insula across every gray matter voxel in an unconstrained fashion. Further, our observations from the rAI seed region were confirmed using a reverse inference method, by seeding the DLPFC region that showed a prominent diagnostic effect. We used fMRI acquisition during a task-free resting state, so that the inferences are not influenced by differences in effort or task performance in patients. Nevertheless, it is possible that there are systematic differences in the resting state achieved by patients compared to controls that could explain the differences noted in the present study. Such differences are difficult to quantify in the fMRI set-up, though existing studies suggest that resting state is likely to be less confounded by diagnostic differences than task fMRI studies in schizophrenia (Whitfield-Gabrieli and Ford, 2012). The labeling of a path coefficient from X to Y as excitatory (or inhibitory) reflects a positive (or negative) sign of the Granger causal coefficient when the BOLD signal in region Y is regressed on the BOLD signal in region X at a preceding point in time. However, increased firing of inhibitory neurons might result in an increase on local blood flow and hence an increase in BOLD signal. Therefore, excitatory and inhibitory Granger casual influences between BOLD time courses do not necessarily correspond directly to excitatory and inhibitory neurotransmission, respectively. As a result, models of neural activity drawn from fMRI BOLD signals must be cautiously interpreted.

It is worth noting that we employed processing speed scores to assess cognitive dysfunction and did not undertake an exhaustive cognitive testing on our patient sample. Studies exploring the cognitive landscape of schizophrenia have demonstrated that a broad cognitive deficit that spans multiple domains of cognition is present in a substantial number of patients (Dickinson et al., 2011). In particular, information-processing speed has emerged as the single most consistent cognitive deficit (Dickinson et al., 2007; Rodríguez-Sánchez et al., 2007). In the future, more detailed exploration of other cognitive domains that are influenced by the salience-execution loop integrity is warranted.

### Possible Confounding Effects of Hemodynamic Delay

Differences in hemodynamic delay between brain regions might in principle confound inferences based on neural delays. In particular, Smith et al. (2011) reported that when GCA was applied to modeled data in which hemodynamic delay varied randomly between subjects, the identification of causal influences was only slightly above chance. However, using hemodynamic responses derived from real data, Schippers et al. (2011) demonstrated that GCA identified causal influences in group studies with good sensitivity and specificity.

When effects are observed using random-effects analysis in which the effect to interests is compared with variance between subjects, the detection of a significant group effect implies the occurrence of a systematic delay in neural and/or hemodynamic response. The results obtained by Schippers et al. (2011) indicate that the effects are most likely to be neural. This conclusion is supported by the fact that the regions involved are served by different arteries and therefore group effects due to hemodynamic delay would only be expected if there were differences in arterial transmission times that were consistent across subjects. However, any such systematic differences would be expected to be similar in the two hemispheres, yet neither the effects reported by Sridharan et al. (2008) nor those that we report are symmetrical across the hemispheres. Furthermore, examination of the timing of regional neural activity using magnetoencephalography (Brookes et al., 2012) demonstrates appreciable neural delays between occipital cortex and insula during various visual tasks, consistent with our present findings that occipital cortex exerts a Granger causal influence on insula.

An additional issue raised by Smith et al. (2011) is the possibility that in a Granger causality analysis, findings might be distorted by zero-lag correlations “bleeding into” the time-lagged relationships. We have demonstrated that significant zero-lag correlations between insula and other brain regions occur at different locations from the Granger causal effects of insula on other brain regions.

To our knowledge, this is the first study to examine time-directed neural primacy effects during task-free resting state in schizophrenia. Our findings extend the neuronal network level models informing the pathophysiology of this illness. Effective cognitive control requires successful suppression of distractors (e.g., spontaneous internal thoughts) but at the same time must be responsive to unexpected stimuli, which though irrelevant to the task are salient for our homeostatic defense (Su et al., 2011). The concept of “proximal salience” refers to the switching between brain states (e.g., task-focused, resting or internally focused, and sensory-processing states) brought on by a momentary state of neural activity within the salience processing system, anchored in the rAI and the dACC (Palaniyappan and Liddle, 2012). We infer that the breakdown of the causal influence to and from the salience processing system in schizophrenia amounts to a failure of proximal salience mechanism. The present study highlights the importance of studying the pathways of failed interaction between large-scale networks in the pathophysiology of schizophrenia. Further, it raises the question of whether the indices of failed integration between the large-scale networks, especially the paralimbic SN and the multimodal CEN, could be employed in prognostic classification and treatment monitoring of patients with psychotic symptoms.

## Experimental Procedures

### Participants

The sample consisted of 38 patients satisfying DSM-IV criteria for schizophrenia or schizoaffective disorder and 35 healthy controls. Patients were recruited from the community-based mental health teams (including Early Intervention in Psychosis teams) in Nottinghamshire and Leicestershire, UK. The diagnosis was made in a clinical consensus meeting in accordance with the procedure of Leckman et al. (1982), using all available information including a review of case files and a standardized clinical interview (SSPI) (Liddle et al., 2002). All patients were in a stable phase of illness (defined as a change of no more than ten points in their Global Assessment of Function [GAF] score, assessed 6 weeks prior and immediately prior to study participation) and the median duration of illness was 6.5 years (range: 1–29 years). We also collected information from case files regarding duration of illness, quantified current occupational and social dysfunction using the Social and Occupational Functioning Assessment Scale (SOFAS) (Goldman et al., 1992), and assessed speed of cognitive processing, a consistent and prominent cognitive deficit in schizophrenia using the Digit Symbol Substitution Test (DSST) (Dickinson et al., 2007). DSST was administered using a written and an oral format with a mean DSST score computed from the two formats (Palaniyappan et al., 2013).

Healthy controls were recruited from the local community via advertisements and included 38 subjects free of any psychiatric or neurological disorder group matched for age and parental socioeconomic status (measured using National Statistics - Socio Economic Classification; Rose and Pevalin, 2003) to the patient group. The study was given ethical approval by the National Research Ethics Committee, Derbyshire, UK. All volunteers gave written informed consent. Additional details on the participants and the fMRI image acquisition are provided in the Supplemental Information.

### fMRI Data Preprocessing

fMRI data was preprocessed using SPM8 (http://www.fil.ion.ucl.ac.uk/spm and Data Processing Assistant for resting-state fMRI; Chao-Gan and Yu-Feng, 2010). Data were corrected for slice-timing differences and spatially realigned to the first image of the data set. Movement parameters were assessed for each participant, and participants were excluded if movement exceeded 3 mm. Further, we employed ArtRepair to correct movement artifacts using an interpolation method (http://cibsr.stanford.edu/tools/human-brain-project/artrepair-software.html). The first five volumes of functional images were discarded to allow stability of the longitudinal magnetization. A single data set was produced from a weighted summation of the dual-echo dynamic time course (Posse et al., 1999). Retrospective physiological correction of this data set was then performed (Glover et al., 2000). The functional scans were then spatially normalized using the unified segmentation approach and smoothed using a Gaussian kernel of 8 mm full-width at half-maximum. After this, linear detrending and filtering using a band-pass filter (0.01–0.08Hz) was done to eliminate low-frequency fluctuations and high-frequency noise. Finally, variance accounted for by nuisance covariates including six head motion parameters, global mean signal, white-matter signal, and CSF signal was removed by regression before conducting a seed-based regional functional connectivity analysis.

### Selection of the Seed Region

As our primary hypothesis was related to the influence of right anterior rAI on the executive system, we determined the anatomic location of the rAI seed using functional activation data during a two-back task performed by all subjects included in the study (one-sample t test, familywise error [FWE] corrected p < 0.05). A 6 mm radius sphere centered on the local maxima (x = 33, y = 21, z = −3) corresponding to the rAI was used as the seed region for further analysis. The location of this seed (Figure S5) corresponds to anterior compartment of the insula that is frequently mapped to the behavioral domains of attentional processing and socioemotional function (Klein et al., 2013).

### Granger Causality

Granger’s principle suggests that a time series (X) exerts a causal influence (termed as Granger causality) on another time series (Y) if the preceding states of X predict the state of Y uniquely, over and above the variance explained by the preceding states of Y itself. In the present study, we estimated (1) X-to-Y effects, the Granger causal effects of the time series of the anterior insula seed region (X) on every other gray matter voxel in the brain (Y), and (2) Y-to-X effects, the Granger causal effect of every other gray matter voxel on the rAI. The path coefficient maps for the Granger causality were generated using a time lag order of 1 (1 TR, 2.5 s). In contrast to Sridharan et al. (2008), we used signed-path coefficients (Hamilton et al., 2011; Zang et al., 2012) instead of F-residuals so as to infer the probable excitatory or inhibitory effects of the directed physiological influences. The path coefficient of +1 from region X to Y in this model suggests that one unit of change in the activity of region X in a specific direction brings a unit change in the activity of region Y in the same direction in the context of Granger causality. We refer to this as excitatory influence. Similarly, a path coefficient of −1 from region X to Y suggests that one unit of change in the activity of region X in a specific direction brings a unit change in the activity of region Y in the opposite direction (we refer to this as inhibitory influence). In contrast to residual-based GCA models in which the net causal flow is calculated by subtracting x-to-y from y-to-x effects, bivariate GCA allows for the physiological possibility that bidirectional influences of opposite effects could simultaneously exist in the brain. Further, the signed-path coefficient maps allow parametric statistical analysis for group-level inference (Hamilton et al., 2011). This helped us to determine the multimodal brain region that showed most significant difference between the patients and controls in the causal influence to and from the rAI.

Bivariate first-order coefficient-based voxelwise GCA was performed using the REST software (http://www.restfmri.net), using Chen’s method of signed-path coefficients.

### Functional Connectivity

To compute FC, we calculated Pearson’s correlation coefficients between the mean time series of the rAI seed region and every voxel in the brain for each subject. Resulting voxelwise correlation coefficients were then converted to produce whole-brain z maps using a Fisher transform for further second-level statistical analyses.

### Statistical Analysis

The FC and GCA maps from each individual subject were analyzed using separate one-sample t test for the entire sample (both patients and controls) with an FWE corrected p < 0.05 for positive and negative coefficients. This threshold was used to ensure that the clusters emerging in the one-sample t test are unlikely to be due to a type 1 error. From the results, we derived search volume masks for the FC and GCA to constrain the subsequent between-group analyses. These masks represented regions with significant instantaneous positive correlation or anticorrelation with the seed region and significant excitatory or inhibitory influence to and from the seed region in the whole sample. Between-group analyses were conducted using an unpaired t test (FWE corrected p < 0.05), with the search volume corrected for the masks used in the analyses. For regions showing significant group differences at the FWE-corrected threshold, follow-up one-sample t tests were conducted to investigate the direction of the Granger causal influence in each group separately. These tests were Bonferroni corrected for a total of eight follow-up comparisons. In addition to such constrained analyses, we also carried out a whole-brain between-group analysis (at uncorrected p < 0.001) in order to identify informative group differences that may exist in regions outside the masks derived from one-sample t tests. As this exploratory search has a higher likelihood of identifying false-positive clusters, we applied an additional extent criterion of k = 30. Age and gender were used as covariates in all group-level analyses. Within the patient group, bivariate correlations were used to examine the influence of antipsychotic medications on the mean coefficients within the clusters that emerged as significant from the two-sample t tests in both FC and GCA comparisons. All group-level analyses were carried out using the SPM8 software and the toolboxes MarsBar (http://marsbar.sourceforge.net) and xjview (http://www.alivelearn.net/xjview8), in addition to MRICron (http://www.mccauslandcenter.sc.edu/mricro/mricron) to visualize the results.

### Mediation Analysis

Mediation analysis was carried out using the Preacher and Hayes model (Preacher and Hayes, 2004), predicting the Granger influence of rAI on the time course of the signal in the DLPFC (dependent variable, DV) from the diagnosis (independent variable, IV). The mediator (M) of this relationship was the first eigenvariate of the functional connectivity between rAI and the clusters showing significant diagnostic effect in the FC analysis. This eigenvariate represented the typical connectivity in each subject between the rAI and each of the voxels showing abnormal FC in schizophrenia. We evaluated the total effect of diagnostic status on the rAI to DLPFC influence and partitioned this effect to the direct effect and the indirect effect mediated by the presence of functional dysconnectivity related to the rAI. A bootstrapping method with 5,000 iterations was used to test the 95% confidence intervals of the indirect effects (Preacher and Hayes, 2008).

### GCA Coefficients and Illness Severity

In the present study, we observed a significant failure of the directed influences within a salience-execution loop comprised of rAI, rDLPFC, and dACC. We also observed a significant failure of directed influence to and from several other brain regions (other than dACC and DLPFC) and the rAI. This includes a reduction in the Granger causal inflow from bilateral visual cortices and right hippocampus to the rAI and from the rAI to precuneus in patients. In light of this, we investigated the relationship between illness severity and these abnormal Granger causal interactions in patients.

SSPI scores on reality distortion, disorganization, and psychomotor poverty, measured on the same day of scanning, provide information regarding the symptom burden that persists despite antipsychotic treatment. In addition, cognitive deficits (reduced DSST score), longer duration of illness, and higher functional disability (reduced SOFAS score) also indicate illness severity. The variables reflecting disease severity (three SSPI scores, duration of illness, DSST score, and SOFAS score) showed significant bivariate relationships (mean of absolute correlation coefficients |r| = 0.34).

The net Granger causal influences (computed as [(x-to-y) – (y-to-x)] coefficients) among the three nodes in the salience-execution loop were highly correlated (|r| = 0.46). Similarly, the Granger causal influences to and from rAI to regions showing the most significant between-group differences (rAI to precuneus, from left and right visual cortex and right hippocampal region to rAI—reported in Table 1) were also correlated with each other (|r| = 0.3). Therefore, we performed three separate principal component analyses to extract first unrotated principal factors explaining the largest proportion of variance in (1) the measures of illness severity, (2) the causal interactions among rAI, rDLPFC, and dACC, and (3) the causal influences to and from rAI to regions showing most significant between-group differences. This data reduction approach reduced the likelihood of type 1 errors occurring due to multiple testing of the relationships among the various neuroimaging and symptom variables.

An “illness severity” factor explaining 40% of variance, a “salience-execution loop” factor explaining 52% of variance, and a “visual inflow” factor explaining 48.5% of variance emerged from this analysis (Table S7). To study the relative contribution of the salience-execution loop factor and the visual inflow factor in predicting the illness severity, we conducted a multiple regression analysis with antipsychotic dose as a covariate. There was no significant colinearity among the independent variables. All variables (covariate and predictors) were entered in a single step in the regression model.

## Figures and Tables

**Table 1:** Two-Sample t Test of the Difference in the Directed Influence to and from the Right Anterior Insula in Patients and Controls

Regions	MNI Coordinates (x, y, z)	Mean (SD) Path Coefficient in Controls	Mean (SD) Path Coefficient in Patients	p value, Peak Intensity, and Cluster Size (k = Voxel Count)
**Causal outflow from the rAI (x-to-y coefficients)**				

Right middle frontal^∗∗^	48, 34, 24 mm	0.103(0.08)	0.027(0.08)	p(SVC) = 0.035, T = 3.85, k = 32 controls > schizophrenia
Left precuneus	−4, −70, 32 mm	0.072 (0.14)	−0.039(0.10)	p(unc.) < 0.001, T = 3.93, k = 72 controls > schizophrenia

**Causal inflow to the rAI from rest of the brain (y-to-x coefficients)**				

Left superior occipital, cuneus (BA18 and BA19)^∗^	−18, −92, 22 mm	0.045(0.05)	−0.035 (0.09)	p(cFWE.) = 0.006, T = 4.74, k = 351 controls > schizophrenia
Right hippocampus and parahippocampal gyrus	32, −8, −16 mm	0.031(0.07)	−0.025(0.05)	p(unc.) < 0.001, T = 3.97, k = 33 controls > schizophrenia
Right superior occipital, cuneus (BA18 and BA19)	24, −90, 16 mm	0.034(0.05)	−0.030 (0.08)	p(unc.) < 0.001, T = 3.91, k = 112 controls > schizophrenia

^∗∗^p(SVC), familywise error corrected within the search volume at p < 0.05. ^∗^p(cFWE), cluster level familywise error corrected at p < 0.05. p(unc.), the clusters observed using a more lenient criteria of p < 0.001 are thresholded using an extent cluster k = 30 in the unconstrained search. See also Figure S1.

**Table 2:** Two-Sample t Test of the Difference in the Directed Influence of Right DLPFC on Rest of the Brain between Patients and Controls

Regions	MNI Coordinates (x, y, z)	Mean (SD) Path Coefficient in Controls	Mean (SD) Path Coefficient in Patients	p Value, Peak Intensity, and Cluster Size (k = Voxel Count)
**Causal outflow from the DLPFC (x-to-y coefficients)**				

Bilateral dorsal anterior cingulate^∗∗^	0, 28, 20 mm	−0.079(0.06)	−0.009(0.06)	p(cFWE) = 0.01, T = −4.91, k = 452 schizophrenia > controls
Left cerebellum posterior lobe/crus	−42, −70, −34 mm	0.075(0.06)	0.018(0.03)	p(unc.) < 0.001, T = 4.74, k = 164 controls > schizophrenia
Right anterior insula and orbitofrontal cortex	34, 26, −14 mm	−0.052(0.06)	0.013(0.07)	p(unc.) < 0.001, T = −4.48, k = 180 schizophrenia > controls
Right inferior frontal operculum	54, 18, 14 mm	−0.059(0.10)	0.022(0.06)	p(unc.) < 0.001, T = −4.23, k = 163 schizophrenia > controls
Left anterior insula and orbitofrontal cortex	−32, 18, −14 mm	−0.041(0.05)	0.018 (0.07)	p(unc.) < 0.001, T = −4.22, k = 146 schizophrenia > controls
Right cerebellum posterior lobe/crus	16, −78, −32 mm	0.053(0.09)	−0.010(0.05)	p(unc.) = < 0.001, T = 3.62, k = 38 controls > schizophrenia

**Causal inflow to the DLPFC from rest of the brain (y-to-x coefficients)**				

Left anterior insula and orbitofrontal cortex^∗∗^	−32, 22, −12 mm	0.046(0.04)	−0.009(0.04)	p(cFWE) < 0.001, T = 5.98, k = 523 controls > schizophrenia
Bilateral dorsal anterior cingulate^∗∗^	2, 18, 30 mm	0.068(0.05)	0.001(0.05)	p(cFWE) = 0.001, T = 5.83, k = 481 controls > schizophrenia
Inferior frontal gyrus^∗∗^	56, 16, 14 mm	0.054(0.05)	−0.008(0.04)	p(cFWE) = 0.022, T = 5.05, k = 243 controls > schizophrenia
Right anterior insula and orbitofrontal cortex^∗∗^	32, 28, −14 mm	0.062(0.05)	−0.007(0.05)	P(cFWE) < 0.001, T = 5.05, k = 539 controls > schizophrenia
Parieto-occipital sulcus and precuneus	−14, −56, 20 mm	−0.040(0.07)	0.026(0.07)	p(unc.) < 0.001, T = 3.97, k = 32 schizophrenia > controls
Supplementary motor area BA6	4, −26, 64 mm	−0.047(0.06)	0.021(0.08)	p(unc.) < 0.001, T = 3.96, k = 70 schizophrenia > controls
Right cerebellum posterior lobe and crus	14, −82, −32 mm	−0.035(0.05)	0.020(0.06)	p(unc.) < 0.001, T = 3.83, k = 37 schizophrenia > controls

^∗∗^p(cFWE), cluster level familywise error corrected at p < 0.05 (cluster inclusion threshold p < 0.001). p(unc.), clusters observed using peak threshold p < 0.001 and an extent threshold k = 30 in the unconstrained search. See also Figure S1.

**Table 3:** Two-Sample t Test of the Difference in the Instantaneous Functional Connectivity of Right Anterior Insula with the Rest of the Brain between Patients and Controls

Regions	MNI Coordinates (x, y, z)	Mean (SD) Correlation Coefficient in Controls	Mean (SD) Correlation Coefficient in Patients	p Value, Peak Intensity, and Cluster Size (k = Voxel Count)
**Controls > schizophrenia**				

Right superior temporal pole^∗∗^	44, 14, −24 mm	0.211(0.17)	0.005(0.13)	p(cFWE) = 0.028, T = 5.09, k = 582
Left superior temporal pole extending to parahippocampal/amygdala^∗∗^	−44, 14, −26 mm	0.094(0.15)	−0.092(0.12)	p(cFWE) = 0.011, T = 4.95, k = 801
Right parahippocampal/amygdala region	36, −10, −22 mm	0.106(0.18)	−0.064(0.15)	p(unc.) < 0.001, T = 4.36, k = 159
Periaqueductal gray matter	6, −24, −18 mm	0.069(0.17)	−0.089(0.18)	p(unc.) < 0.001, T = 3.74, k = 60

**Schizophrenia > controls**				

Left middle frontal	−34, 30, 36 mm	−0.186(0.19)	0.0117(0.18)	p(unc.) < 0.001, T = −4.67, k = 285
Right posterior insula	42, −12, 18 mm	0.017(0.14)ns	0.165(0.18)	p(unc.) < 0.001, T = −3.91, k = 52

^∗∗^p(cFWE), cluster level familywise error corrected at p < 0.05 (cluster inclusion threshold p < 0.001). p(unc.), clusters observed using peak threshold p < 0.001 and an extent threshold k = 30 in the unconstrained search. See also Figure S2 and Table S4.

## Supplemental Information

Document S1. Supplemental Experimental Procedures, Figures S1–S5, and Tables S1–S7
