# Supplementary material for: Neural Primacy of the Salience Processing System in Schizophrenia
Source: Neuron. 2013 Aug 21;79(4):814–28. doi: 10.1016/j.neuron.2013.06.027 (PMC3752973; doi:10.1016/j.neuron.2013.06.027)
Supplement: Document S1. Supplemental Experimental Procedures, Figures S1–S5, and Tables S1–S7 [file mmc1.pdf]

Neuron, Volume *79*

Supplemental Information

Neural Primacy of the Salience

Processing System in Schizophrenia

Lena Palaniyappan, Molly Simmonite, Thomas P. White, Elizabeth B. Liddle, and Peter F. Liddle

SUPPLEMENTAL FIGURES

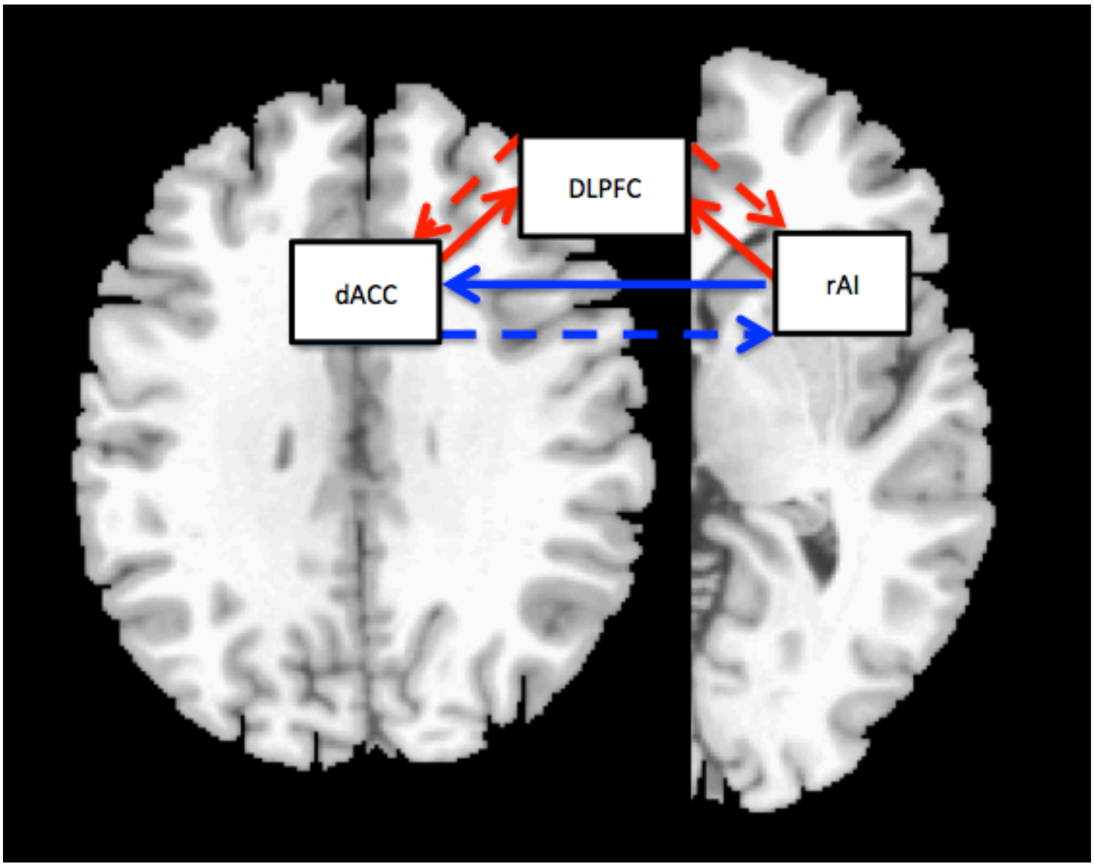

Figure S1 (Related to Table 2): Summary of the Granger causal inferences. Right anterior insula (rAI) has excitatory influence on both dorsal anterior cingulate cortex (dACC) and dorsolateral prefrontal cortex (DLPFC); in turn both of these regions inhibit the rAI in controls. Solid lines represent a positive Granger influence; dashed lines represent a negative Granger influence. Lines drawn in red indicate the pathways that show significant abnormalities in schizophrenia when compared to controls. The interaction between the DLPFC and both nodes of the Salience Network (rAI and dACC) are affected in schizophrenia. The rAI to dACC loop is relatively intact in schizophrenia.

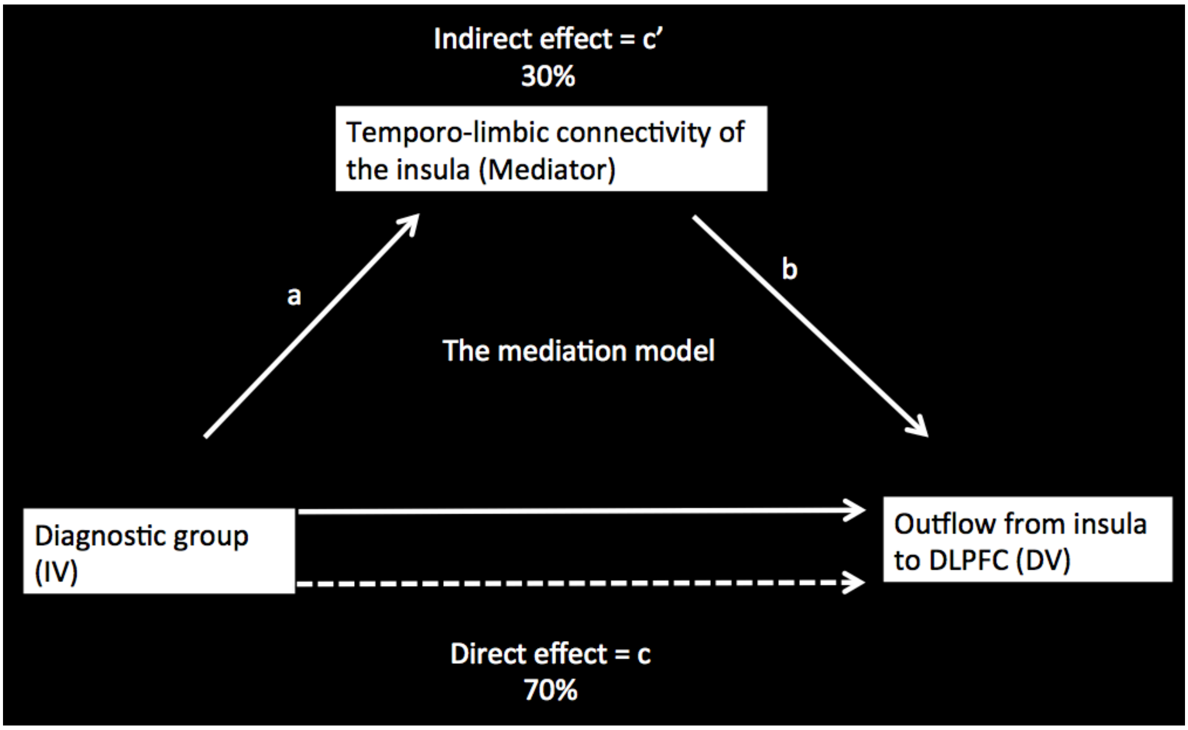

Figure S2 (Related to Table 3): The mediation model. A single mediation model with the first eigenvariate of functional connectivity between the right anterior insula and the temporolimbic clusters (bilateral temporal pole, hippocampal formation and periaqueductal grey) as a mediator of the relationship between diagnostic status and the directed influence (x to y) from right anterior insula to right DLPFC. IV: independent variable, DV: dependent variable.

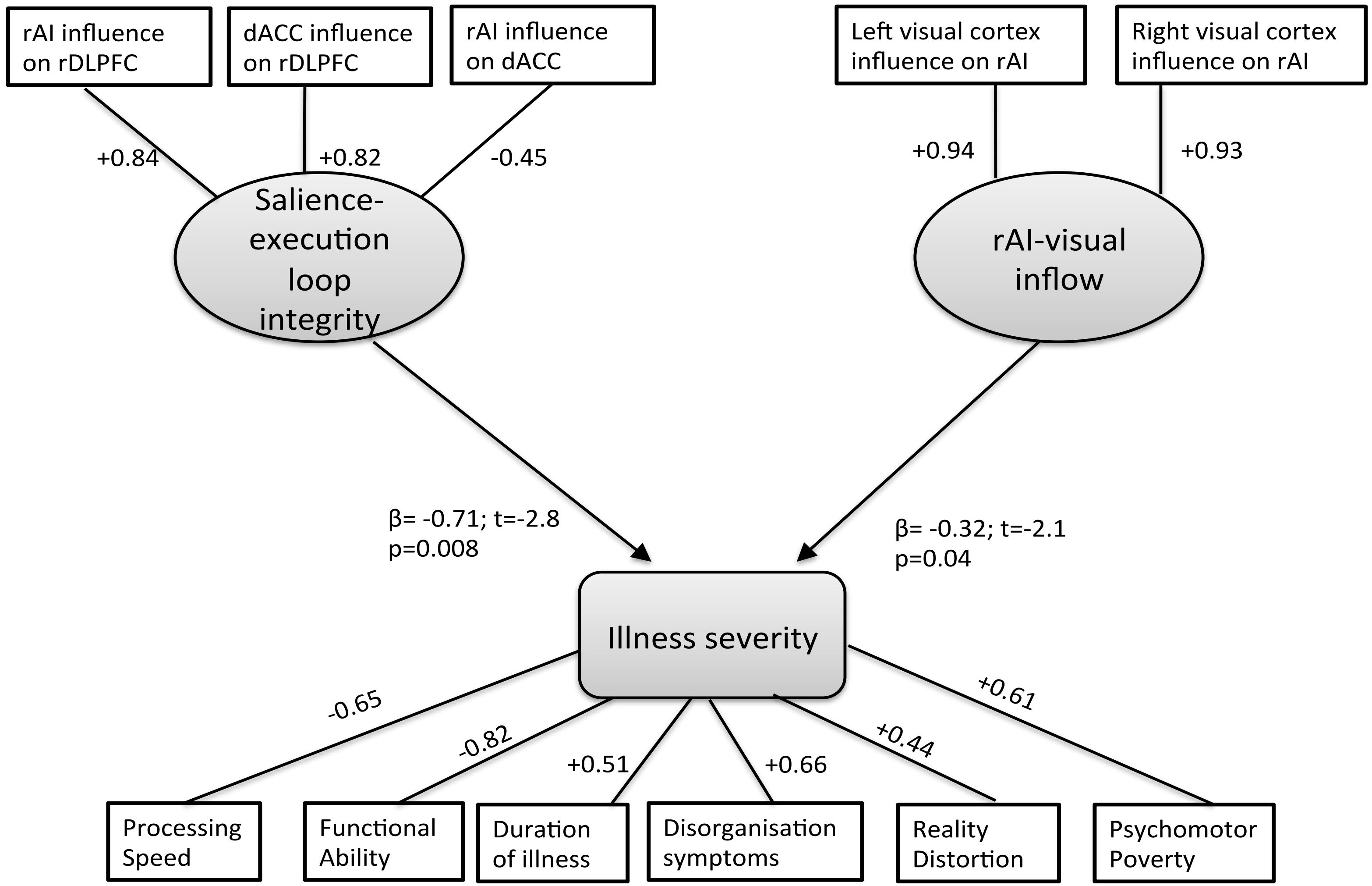
rAI influence on rDLPFC
dACC influence on rDLPFC
rAI influence on dACC
Left visual cortex influence on rAI
Right visual cortex influence on rAI
+0.84
+0.82
-0.45
+0.94
+0.93
Salience-execution loop integrity
rAI-visual inflow
β= -0.71; t=-2.8 p=0.008
β= -0.32; t=-2.1 p=0.04
Illness severity
-0.65
-0.82
+0.51
+0.66
+0.44
+0.61
Processing Speed
Functional Ability
Duration of illness
Disorganisation symptoms
Reality Distortion
Psychomotor Poverty

Figure S3 (related to Figure 2). Relationship between illness severity, salience-executive loop integrity and visual inflow to right anterior insula. Variables with loading >0.4 in each of the first unrotated principal factor are shown as components in the figure. Factor scores are indicated using appropriate positive or negative sign. The β, t and p values are parameter statistics derived from a multiple regression analysis to predict the illness severity factor from the salience-executive loop and the visual to rAI inflow factors. rAI: right anterior insula, DLPFC: dorsolateral prefrontal cortex; dACC: dorsal anterior cingulate cortex.

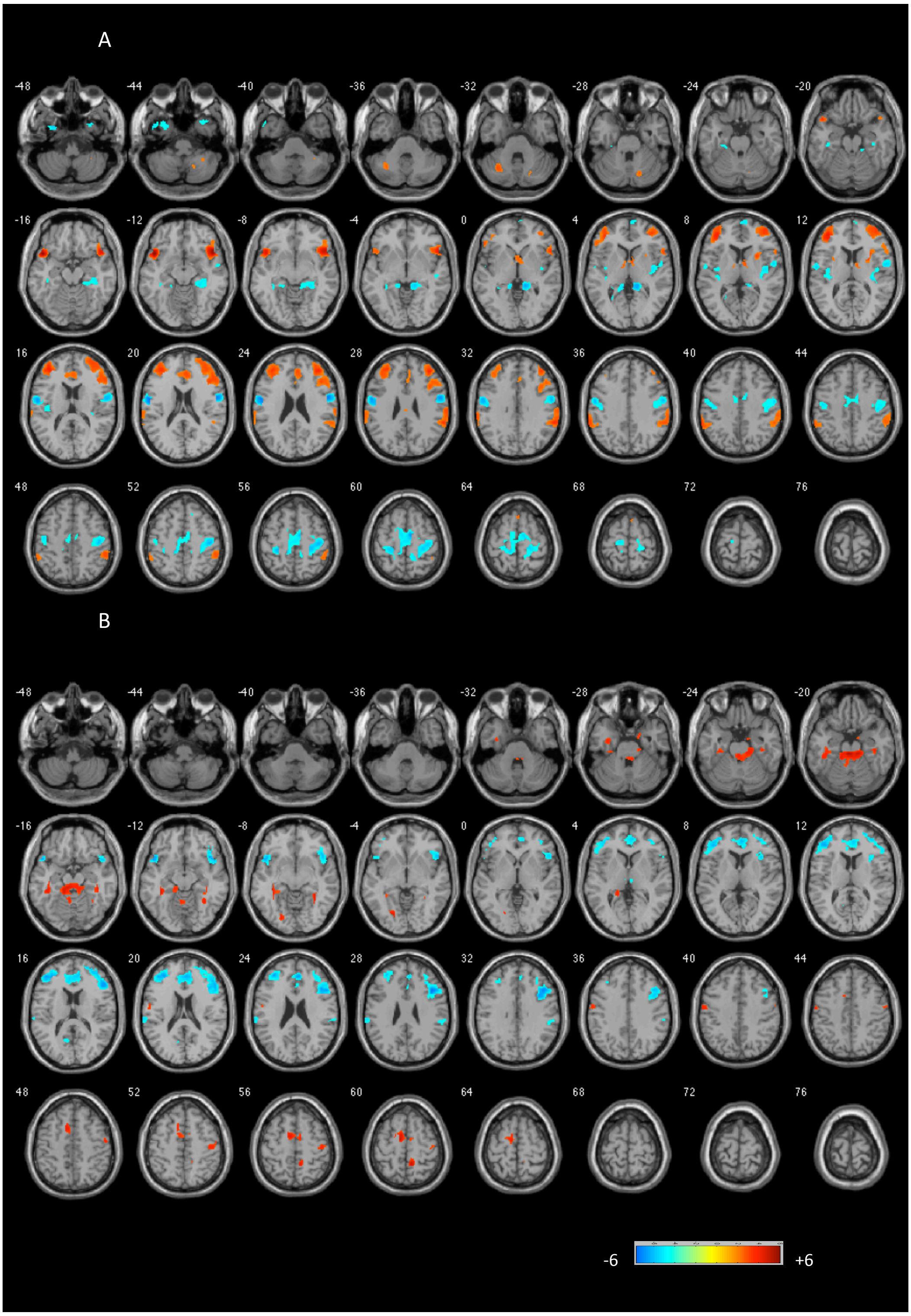

Figure S4 (Related to Figure 1): Granger causal influence to and from the left anterior insula. Top panel A depicts the influence of left anterior insula on the rest of the brain (x to y). Bottom panel B depicts the influence of rest of the brain on

left anterior insula (y to x). The figures show the results of the one sample T test of GCA maps on all subjects (patients and controls). Illustrations drawn on a single subject structural image showing axial slices using xjview at $p<0.001$ uncorrected, k=30. Color bar shows a scale of T values. Warm colours suggest excitatory influence, while cold colours suggest inhibitory influence.

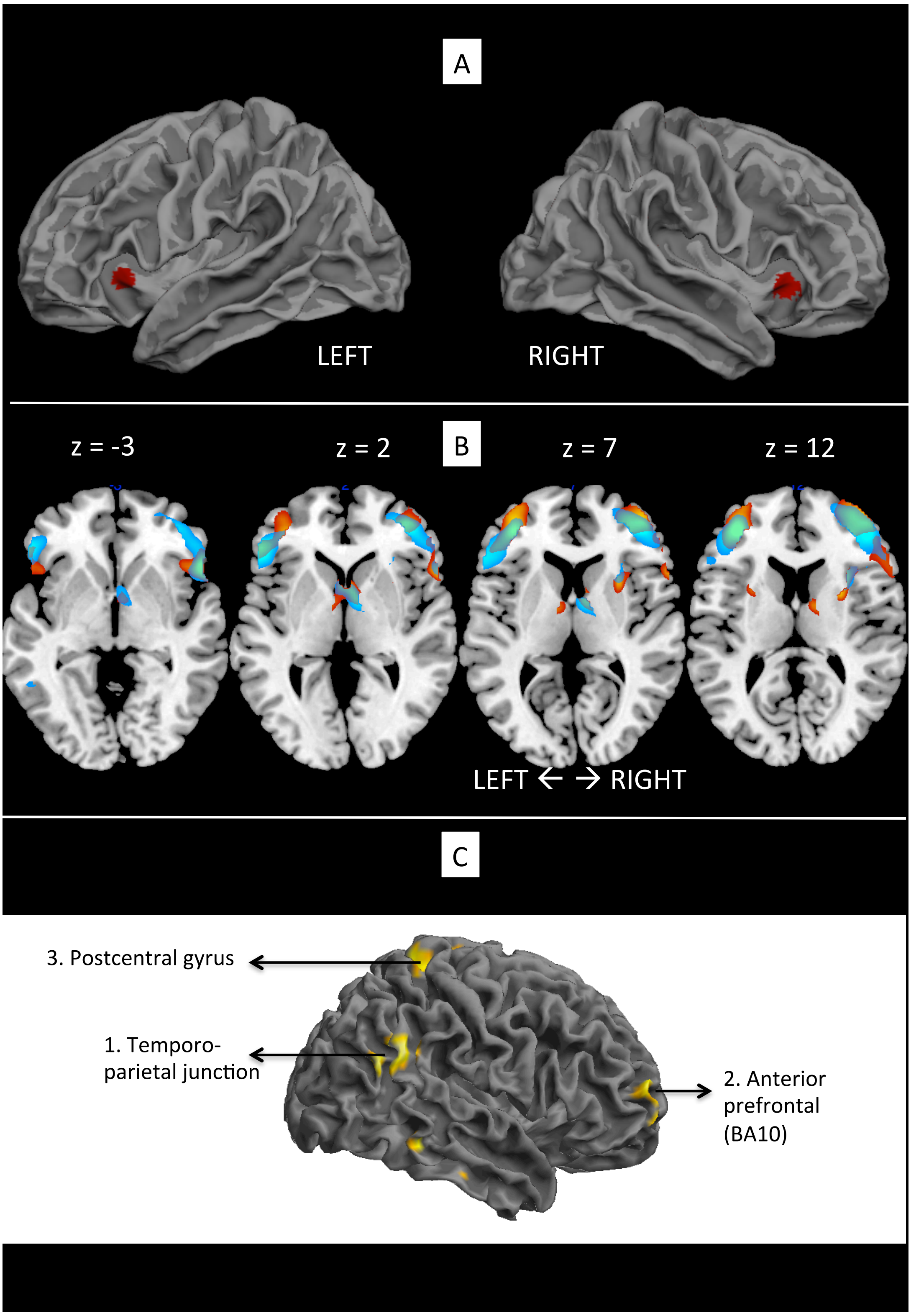

Figure S5 (Related to Supplemental Experimental Procedures): Hemispheric lateralisation of Granger causal influences from anterior insula. A: Right (x=33,y=21,z=-3) and Left (x=-33,y=21,z=-3) anterior insula seed regions

projected on a reconstructed average white matter surface (fsaverage) using Freesurfer software. B. Spatial overlap of causal influence from right (blue-cyan) and left (red-yellow) anterior insula seeds. Each overlay is derived from the one sample T test of the corresponding seed based GCA maps on all subjects (patients and controls). Illustrations drawn on a single subject structural image showing axial slices using MRICron at $p<0.001$ uncorrected, k=30. Slices selected for the best display of lateral prefrontal cortex. C. Differences in the Granger causal (x to y) influence between right and left anterior insula using a paired t test in patients with schizophrenia (n=38). Three clusters showing greater causal influence from right (>left) anterior insula emerged in patients. 1. Temporo-parietal junction (cluster p_{FWE}=0.006; k=431; T=5.74, peak (x,y,z) = 56,-56,30) 2. Anterior prefrontal (BA10) (cluster p_{FWE}=0.042; k=268; T=5.17, peak (x,y,z) = 34,64,-6) 3. Postcentral gyrus (cluster p_{FWE}=0.021; k=324; T=4.63, peak (x,y,z) = 12,-40,64). There were no regions showing excessive casual influence from left (>right) anterior insula seed. No differences in the GCA maps between the right and left anterior insula were notable in healthy controls**.**

SUPPLEMENTAL TABLES

Table S1: Demographic and clinical features (Related to Supplemental Experimental Procedures)

	Patients with schizophrenia	Healthy controls	t/$\chi 2$
**Number**	38	35	-
**Gender (male/female)**	29/9	25/10	$\chi 2=0.23$, p=0.63
**Handedness (right/left)**	33/5	31/4	$\chi 2=0.05$, p=0.82
**Age in years (SD)**	34.5(9.1)	33.5(9.1)	t=0.46, p=0.65
**Mean parental NS-SEC (SD)**	2.4(1.5)	2.1(1.3)	t=0.79, p=0.43
**Mean DSST score**	42.4(10.0)	57.4(9.4)	t=6.5, p<0.0001
**Mean SOFAS score**	53.9(12.8)	-	
**Median duration of illness in years (range)**	6.5(28)	-	
**Median Defined Daily Dose of antipsychotics (range)**	1.0(5.57)	-	-
**Mean total SSPI score**	12.1(7.3)	-	-
**Reality Distortion**	2.26(2.6)	-	-
**Psychomotor Poverty**	2.84(3.5)	-	-
**Disorganisation**	1.39(1.3)	-	-

DSST – Digit Symbol Substitution Test; NS-SEC: National Statistics – Socio Economic Status; SD: standard deviation; SOFAS – Social and Occupational Functioning Assessment Scale; SSPI – Symptoms and Signs of Psychotic Illness. The total SSPI score can vary between 0 and 80. Reality distortion (delusions and hallucinations) can vary between 0 and 8. Psychomotor poverty (anhedonia, underactivity, poverty of speech and flat affect) can vary between 0 and 16. Disorganisation (inappropriate affect, disordered thought form and poor attention) can vary between 0 and 12.

Table S2 (Related to Table 1): One sample T test (patients and controls) of the directed causal influence to and from right anterior insula

Regions	MNI coordinates (x, y, z) in mm	Mean (SD) path coefficient in controls	Mean (SD) path coefficient in patients	Peak intensity (T) and cluster size (k=voxel count) §
Regions positively influenced by rAI				
Right inferior parietal (supramarginal, BA40)	50,-44,52	0.071(0.07)	0.086(0.08)	T=8.15,k=784
Right middle and inferior frontal (BA10,BA46)	38,40,24	0.095(0.07)	0.058(0.06)	T=7.75,k=900
Left cerebellum crus	-30,-64,-32	0.070(0.08)	0.039(0.06)	T=6.33,k=79
Left inferior parietal (supramarginal, BA40)	-52,-42,38	0.058(0.07)	0.066(0.08)	T= 6.18 ,k=321
Left inferior frontal	-52,34,0	0.071(0.10)	0.058(0.08)	T=6.00 ,k=47
Right orbitofrontal and superior temporal	48,16,-12	0.114(0.18)	0.114(0.15)	T=5.72 ,k=38
Left inferior and middle frontal	-36,44,12	0.104(0.12)	0.045(0.08)	T=5.55 ,k=34
Regions negatively influenced by rAI				
Supplementary Motor Area (left and right)	10,-8,56	-0.054(0.05)	-0.039(0.05)	T= 7.57 ,k=383
Left Precentral and Postcentral	-58,-10,22	-0.077(0.07)	-0.047(0.06)	T=7.52 ,k=526
Right Precentral and Postcentral	62,0,22	-0.068(0.07)	-0.037(0.05)	T= 6.93 ,k=276
Right posterior insula	42,-8,6	-0.067(0.08)	-0.040(0.05)	T=6.45 ,k=89
Left Precentral and Postcentral	-22,-30,60	-0.033(0.05)	-0.034(0.04)	T= 5.67 ,k=37
Regions exerting positive influence on rAI				
Left Supplementary motor area, ACC	-10,2,50	0.059(0.07)	0.034(0.04)	T=6.54, k=55
Right Supplementary motor area, ACC	10,-6,56	0.060(0.08)	0.049(0.06)	T=6.65,k=31
Regions exerting negative influence on rAI				
Left middle and inferior frontal	-34,40,16	-0.052(0.06)	-0.025 (0.04)	T=6.28,k=120
Right middle frontal (BA10, BA46)	36,50,16	-0.054(0.06)	-0.031(0.04)	T=6.25 ,k= 307
Left superior medial frontal, ACC	2,44,26	-0.031(0.04)	-0.024 (0.03)	T=5.92 ,k=34
Left inferior frontal	-54,28,8	-0.034(0.06)	-0.036 (0.04)	T=5.82 ,k= 50
Right inferior frontal	44,24,26	-0.042(0.05)	-0.033(0.05)	T=5.77 ,k= 30
§ Familywise Error corrected p<0.05, k=30				

Table S3 (Related to Table 2): One sample T test (patients and controls) of the directed causal influence to and from right DLPFC

**Regions**	**MNI coordinates (x, y, z) in mm**	**Mean (SD) path coefficient in controls**	**Mean (SD) path coefficient in patients**	**Peak intensity (T) and cluster size (k=voxel count) §**
Regions positively influenced by rDLPFC				
Left cerebellum posterior lobe/crus	-44 -70 -38	0.078(0.06)	0.020(0.04)	T=6.67 k=111
Right middle frontal gyrus	44 36 34	0.091(0.07)	0.056(0.08)	T=6.45, k=140
Right superior parietal lobule and angular gyrus	42,-66,50	0.090(0.10)	0.060(0.08)	T=6.13, k=280
Left superior parietal lobule	-26,-68,56	0.081(0.10)	0.050(0.10)	T=5.7 k=44
Regions negatively influenced by rDLPFC				
Right insula, superior temporal gyrus and parietal operculum	36 -24 16	-0.044(0.05)	-0.035(0.04)	T=6.94,K=170
Left insula	-34 -14 14	-0.041(0.04)	-0.028(0.04)	T=5.94 k=39
Right posterior insula	44,12,-12	-0.070(0.07)	-0.046(0.09)	T=5.56, k=69
Regions exerting positive influence on rDLPFC				
Anterior midcingulate	2,14,42	0.071(0.06)	0.023(0.06)	T=6.05,k=76
Right anterior insula	34,22,0	0.072(0.06)	0.021(0.06)	T=6.02, k=35
Regions exerting negative influence on rDLPFC				
Left angular gyrus (inferior parietal lobe)	-46,66,38	-0.043(0.06)	-0.029(0.05)	T=5.25,k=31
§ Familywise Error corrected p<0.05, k=30				

Table S4 (Related to Table 3): One sample T test (patients and controls) of intrinsic functional connectivity between right anterior insula and rest of the brain

Regions	Peak MNI coordinates (x, y, z) in mm	Mean (SD) correlation coefficient in controls*	Mean (SD) correlation coefficient in patients*	Peak intensity (T) and cluster size (k=voxel count)§
Regions showing positive correlation				
Bilateral anterior insula, extending to anterior and midcingulate, bilateral inferior frontal, middle frontal and superior temporal gyrus, supramarginal gyrus, putamen and thalamus	32,20,0<br>-32,20, -2<br>4,12,46	0.257(0.07)	0.249(0.08)	T=82.2, k=31948
Right middle temporal gyrus	54, -52, -8	0.151(0.17)	0.130(0.16)	T=8.0, k=124
Right anterior midcingulate	12,-28,36	0.167(0.16)	0.135(0.20)	T=8.0, k=80
Left precuneus	-16, -74,30	0.111(0.13)	0.123(0.14)	T=7.6, k=161
Left anterior midcingulate	-12,-28,36	0.121(0.14)	0.140(0.19)	T=7.1, k=137
Right precuneus	18, -70,34	0.137(0.16)	0.120(0.19)	T=6.2, k=138
Left inferior parietal	-34,-44,38	0.122(0.16)	0.125(0.19)	T=6.1, k=50
Regions showing negative correlation				
Right inferior temporal, fusiform and parahippocampal region	60, -6 -28	-0.164(0.09)	-0.199(0.11)	T=14.2, k=4044
Right posterior cingulate / precuneus	2, -58,22	-0.236(0.12)	-0.189(0.12)	T=13.9, k=2018
Left inferior temporal gyrus, fusiform and parahippocampal region	-58, -2 -34	-0.175(0.06)	-0.172(0.10)	T=13.4, k=11792
Left angular gyrus	-44, -70, 40	-0.253(0.11)	-0.201(0.17)	T=13.1, k=2181
Bilateral ventral and superior medial prefrontal	-38,16,56	-0.229(0.13)	-0.186(0.17)	T=12.4, k=5373
Right angular gyrus	58, -66,26	-0.215(0.14)	-0.182(0.18)	T=11.8, k=1239
Bilateral paracentral lobule	-2, -34,72	-0.127(0.12)	-0.146(0.16)	T=10.9, k=1185
Posterior lobe and tonsil of cerebellum	4, -54,-50	-0.167(0.16)	-0.119(0.15)	T=9.3, k=297
Left medial frontal gyrus	-4,14,-26	-0.097(0.17)	-0.183(0.18)	T=7.53, k=77
Left orbitofrontal cortex	-44, 32 -18	-0.134(0.14)	-0.150(0.18)	T=6.5, k=126
Left precentral gyrus	-36,-24,62	-0.105(0.15)	-0.115(0.16)	T=6.38,k=126
Right precentral gyrus	40, -22,66	-0.099(0.15)	-0.131(0.17)	T=6.3, k=96
* Values are z transformed. § Voxel level familywise error corrected p<0.05, k=30				

Table S5 (Related to Supplemental Experimental Procedures): One sample T test (patients and controls) of the directed causal influence to and from left anterior insula

Regions	MNI coordinates (x, y, z) in mm	Mean (SD) path coefficient in controls	Mean (SD) path coefficient in patients	Peak intensity (T) and cluster size (k=voxel count) §
Regions positively influenced by left AI				
Left superior temporal and orbitofrontal gyrus	-46,17,-13	0.107(0.11)	0.069(0.08)	T=7.6, k=195
Right inferior orbitofrontal / insula	48,20,-10	0.080(0.09)	0.069(0.08)	T=7.3, k=232
Left middle frontal gyrus	-37,46,14	0.077(0.11)	0.040(0.06)	T=5.6, k=196
Right middle frontal gyrus	39,43,26	0.060(0.08)	0.030(0.07)	T=5.1, k=50
Regions negatively influenced by left AI				
Left precentral gyrus	-56,-5,24	-0.063(0.08)	-0.024(0.04)	T=6.5, k=263
Right precentral gyrus	58,-3,24	-0.058(0.07)	-0.020(0.04)	T=6.3, k=194
Supplemental Motor Area	9,-8,60	-0.031(0.04)	-0.030(0.05)	T=5.7, k=48
Right precuneus	14,-37,2	-0.058(0.06)	-0.030(0.07)	T=5.6, k=73
Right postcentral gyrus	38,-24,55	-0.052(0.08)	-0.023(0.04)	T=5.1, k=110
Regions exerting positive influence on left AI				
Anterior lobe of cerebellum	1,-30,-19	0.036(0.05)	0.035(0.06)	T=5.4, k=163
Left Supplementary motor area	-8,-3,60	0.053(0.09)	0.061(0.09)	T=4.9, k=48
Regions exerting negative influence on left AI				
Left superior temporal pole	-47,17,-11	-0.035(0.05)	-0.33(0.05)	T=5.9, k=67
Right middle and inferior frontal	46,24,25	-0.040(0.03)	-0.034(0.05)	T=5.9, k=459
Right inferior frontal gyrus	48,20,-9	-0.029(0.05)	-0.036(0.04)	T=5.6, k=70
Left middle frontal gyrus	-37,42,16	-0.047(0.05)	-0.30(0.05)	T=5.3, k=254
Right anterior cingulate	6,43,17	-0.026(0.05)	-0.044(0.05)	T=5.2, k=133
§ Familywise Error corrected p<0.05, k=30				

Table S6 (Related to Supplemental Experimental Procedures): Two-sample T test of the difference in the directed influence to and from the left anterior insula in patients and controls.

Regions	MNI coordinates (x, y, z) in mm	Mean (SD) path coefficient in controls	Mean (SD) path coefficient in patients	Peak intensity and cluster size (k = voxel count), P(unc.)<0.001
Causal outflow from the left AI (x to y coefficients)				
Left precentral gyrus	-55,-7,25	-0.082(0.09)	-0.019(0.05)	T= 3.89 , k= 49 Schizophrenia>Controls
Right medial frontal gyrus (right rectus)	6,55,-15	-0.074(0.14)	0.020(0.07)	T= 3.81 , k= 69 Schizophrenia>Controls
Left medial frontal gyrus (left rectus)	-9,49,17	-0.055(0.10)	0.015(0.06)	T= 3.79 , k= 31 Schizophrenia>Controls
Right precentral and postcentral	64,-2,18	-0.06(0.07)	-0.004(-.05)	T=3.76 , k= 49 Schizophrenia>Controls
Right superior medial frontal	9,61,7	-0.06(0.09)	0.014(0.06)	T=3.64 , k= 109 Schizophrenia>Controls
Causal inflow to the left AI from rest of the brain (y to x coefficients)				
Left middle occipital gyrus (BA19)	-30,-96,12	0.063(0.09)	-0.045 (0.14)	T= 3.96 , k=37 Controls> Schizophrenia
P(unc.) clusters observed using a peak threshold p<0.001 and cluster extent threshold k=30 in the unconstrained search. None of the clusters survived FWE corrected p<0.05 for multiple testing.				

Table S7 (Related to Figure 2): Principal components (first unrotated factors) relating to illness severity, salience-execution loop integrity and visual inflow to rAI in patients with schizophrenia

Variables	Factor loading	Interpretation of the principal factor based on major components (variables with loading >0.4)
Illness severity (40% of variance)		Higher factor scores seen in patients with poor functional ability, poor processing speed and higher burden of disorganisation, psychomotor poverty and reality distortion.
Social and occupational functioning (SOFAS) score	-0.82	
Disorganisation score (SSPI)	+0.66	
Processing speed score (DSST)	-0.65	
Psychomotor Poverty score (SSPI)	+0.61	
Duration of illness	+0.51	
Reality Distortion score (SSPI)	+0.44	
Causal influence within the salience-execution loop (53% of variance)		Higher factor scores seen in patients with higher excitatory influence from rAI and dACC to DLPFC, and reduced excitatory influence from rAI to dACC. (In the present sample, this pattern is observable in healthy controls and is suggestive of normal physiological integrity of the salience-execution loop)
rAI to rDLPFC influence	+0.84	
dACC to rDLPFC influence	+0.82	
rAI to dACC influence	-0.45	
Visual inflow to rAI (48.5% of variance)		Higher factor scores seen in patients with higher excitatory influence from visual cortex to rAI. (In the present sample, this pattern is observable in healthy controls and is suggestive of normal physiological integrity of the visual input to rAI)
Left visual cortex to rAI influence	+0.94	
Right visual cortex to rAI influence	+0.93	
Right hippocampus to rAI influence	+0.35	
rAI to precuneus influence	+0.28	

rAI: right anterior insula, rDLPFC: right Dorsolateral prefrontal cortex, dACC: dorsal anterior cingulate cortex, SSPI: Signs and Symptoms of Psychotic Illness, DSST: Modified Digit Symbol Substitution Test, SOFAS: Social and Occupational Functioning Assessment Scale.

Supplemental experimental procedures

Participants

Subjects with age <18 or >50, subjects with neurological disorders, current substance dependence, or IQ < 70 estimated using Quick Test (1) were excluded. 36 out of 38 patients were receiving treatment with antipsychotics (clozapine n=5, other atypical antipsychotics n=25, typical neuroleptics n=5, combined typical and atypical agents n=1) and had no change in their prescriptions for the 6 weeks preceding the scan. The median Defined Daily Dose (WHO Collaborating Centre for Drug Statistics and Methodology, 2003) of antipsychotics was 1 (range from 0 to 5.6). Patients with schizophrenia were interviewed on the same day as the scan and symptom scores assigned according to the SSPI.

Controls had similar exclusion criteria to patients; in addition subjects with personal or family history of psychotic illness were excluded. A clinical interview by a research psychiatrist was employed to ensure that the controls were free from current axis 1 disorder and history of either psychotic illness or neurological disorder.

Excluded subjects

The original sample consisted of 42 patients and 40 controls, but (4 patients, 5 controls) subjects were excluded due to due to movement artefacts, while 1 patient did not complete the image acquisition protocol as planned. There were no differences in the duration of illness (median (range) in the excluded group=

5.5(9), median (range) in the included group= 6.50 (28), p=0.61), symptom severity (mean (SD) in the included group= 12.0(7.3), excluded group= 10.3(7.5), p=0.64) or antipsychotic dose (median (range) in the excluded group= 1.17 (1.25), median (range) in the included group= 1.0 (5.6), p=0.61) between patients who were included or excluded in the analysis.

Image acquisition protocol

Blood oxygenation level-dependent (BOLD) fMRI datasets were acquired on a 3 Tesla Philips Achieva MRI scanner (Philips, Netherlands) during 10 minutes of rest, with eyes open. To enhance sensitivity, dual-echo gradient-echo echo-planar images (GE-EPI) were acquired (2), using an eight-channel SENSE head coil with SENSE factor 2 in anterior-posterior direction, TE1/TE2 25/53 ms, flip angle 85°, 255 x 255 mm field of view, with an in-plane resolution of 3 mm x 3 mm and a slice thickness of 4 mm, and TR of 2500 ms. At each dynamic time point a volume dataset was acquired consisting of 40 contiguous axial slices acquired in descending order. 240 time points were acquired during the resting fMRI paradigm. A magnetisation prepared rapid acquisition gradient echo image with 1 mm isotropic resolution, 256 x 256 x160 matrix, TR/TE 8.1/3.7 ms, shot interval 3 s, flip angle 8°, SENSE factor 2 was also acquired for each participant for image registration.

Granger Causal Analysis of Left Anterior Insula

A number of previous observations suggest that the right anterior insula is a major node in the Salience Network and plays a crucial role in the interactions between large-scale networks (3–5). Studies examining the resting state connectivity of right and left anterior insula (AI) often observe a significant overlap, though the pattern of connectivity related to the Salience Network is strongly right lateralized (6; 7). To explore the causal interactions from the left AI, we repeated the GCA analysis (described in the Methods section of the manuscript) using a left AI seed. A 6-mm radius sphere centered on the local maxima (x=-33, y=21, z=-3) of functional activation corresponding to the left AI during a 2-back task performed by all subjects was used as the left AI seed region.

One sample t test of GCA maps from left AI

In the entire sample (patients and controls, one-sample t test), left AI exerted a significant excitatory influence on the bilateral DLPFC and bilateral inferior orbitofrontal cortex. Significant inhibitory influence of the left AI was noted at bilateral supplementary motor region, precuneus and bilateral precentral regions. Bilateral DLPFC in turn had a significant inhibitory influence on the left AI. In addition, dACC also had significant inhibitory influence on the left AI. Overall, this pattern of causal influence was very similar to those observed using rAI seed. These results are shown in Figure S4 and Table S5.

Between-groups comparison of GCA maps from left AI

Between group (Controls vs. Schizophrenia) analysis conducted using two-sample t-test (FWE corrected $p < 0.05$), with the search volume corrected for the

mask derived from one sample t test did not reveal any significant differences in the causal influences to and from left AI. At an uncorrected peak height threshold $p<0.001$ and cluster extent threshold $k=30$, patients showed a reduction in the inhibitory influence from the left AI to bilateral medial frontal gyrus and precentral gyrus. Similar to the findings from the rAI seed, patients also showed a reduction in the excitatory influence from visual cortex to the left AI, at this uncorrected threshold ($p<0.001$, cluster extent $k=30$). Overall, this suggests that the abnormalities in the causal influence from the anterior insula in general, and the salience-execution loop (AI to DLPFC feedback) in particular, more prominently involve the right hemisphere. More details are presented in Table S6.

Spatial similarity analysis

To determine the degree of topographical overlap between the Granger causal outflow maps from the right and the left AI across the entire sample, we undertook a spatial similarity analysis using the masks derived from the one-sample t tests of right and left AI (x-to-y) GCA maps. Firstly, we derived an intersection (overlap) mask and a combination (union) mask for the two original masks derived from the one-sample t tests. We then calculated the Dice-coefficient of similarity (DCS) using the intersection and the combination masks (8). Conjunction measures such as DCS provide more reliable results when every signal of interest is included in the individual contrasts (9). To enable this, we used an uncorrected threshold of $p=0.001$ when extracting the intersection and the combination masks. A DCS value of 100% means that the both maps have perfect spatial agreement in the distribution of causal influences across the brain.

The DCS test revealed 73% overlap in the topography of causal influences from right and left AI in the entire sample. Of the 27% observed dissimilarity, voxels present in the right AI GCA maps but not in the left AI maps contributed to 21.2% (i.e. 78.5% of the total dissimilarity), with the remaining 5.8% arising from voxels present in the left, but not in the right AI GCA maps. Examination of the conjunction maps revealed that despite the similar distribution of the clusters in both maps, rAI had a larger spatial extent of causal influence (no. of voxels) than the left AI in these clusters (Figure S5).

Group specific differences between the right and left AI GCA maps

A paired t test between the x to y rAI seed maps and left AI seed maps across the entire sample did not reveal any regions of significant differences (at uncorrected $p<0.001$, cluster extent = 30). Interestingly, when the paired t test was restricted within the patient group, three significant clusters with higher Granger causal influence from the right AI compared to the left AI were noted (Figure S5). There were no differences between the left and right AI maps in the healthy controls.

Right anterior portion of the temporoparietal junction (TPJ), along with right anterior insula and anterior prefrontal cortex (aPFC), participates in a ventral attention system associated with the function of orienting to salient external stimuli (10; 11). In patients with schizophrenia, there is a stronger right lateralization of the causal influence from the anterior insula to right TPJ and aPFC, while the causal influence of right AI on the right DLPFC is significantly reduced when compared to healthy controls. Our results suggest that in schizophrenia, a possible imbalance between the bottom-up stimulus processing circuitry and top-down cognitive control circuitry exists at the level of right anterior

insula. This phenomenon of putative stimulus processing imbalance is likely to explain a number of cognitive deficits noted in schizophrenia (12), and requires further experimental exploration using cognitive tasks that modulate stimulus saliency.

References

1. Ammons RB, Ammons CH: The Quick Test (QT): Provisional manual. Psychological Reports 1962; 11:111–161

2. Posse S, Wiese S, Gembris D, Mathiak K, Kessler C, Grosse-Ruyken ML, Elghahwagi B, Richards T, Dager SR, Kiselev VG: Enhancement of BOLD-contrast sensitivity by single-shot multi-echo functional MR imaging. Magn Reson Med 1999; 42:87–97[cited 2012 Jul 7]

3. Sridharan D, Levitin DJ, Menon V: A critical role for the right fronto-insular cortex in switching between central-executive and default-mode networks. Proceedings of the National Academy of Sciences 2008; 105:12569–12574[cited 2009 Aug 31]

4. Supekar K, Menon V: Developmental maturation of dynamic causal control signals in higher-order cognition: a neurocognitive network model. PLoS Comput. Biol. 2012; 8:e1002374[cited 2012 Jun 1]

5. Seeley WW, Menon V, Schatzberg AF, Keller J, Glover GH, Kenna H, Reiss AL, Greicius MD: Dissociable Intrinsic Connectivity Networks for Salience Processing and Executive Control. J. Neurosci. 2007; 27:2349–2356[cited 2010 Jan 15]

6. Cauda F, Costa T, Torta DME, Sacco K, D'Agata F, Duca S, Geminiani G, Fox PT, Vercelli A: Meta-analytic clustering of the insular cortex: Characterizing the meta-analytic connectivity of the insula when involved in active tasks. NeuroImage 2012; 62:343–355[cited 2012 Jun 1]

7. Cauda F, D'Agata F, Sacco K, Duca S, Geminiani G, Vercelli A: Functional connectivity of the insula in the resting brain. Neuroimage 2011; 55:8–23

8. Zou KH, Warfield SK, Bharatha A, Tempany CMC, Kaus MR, Haker SJ, Wells WM, Jolesz FA, Kikinis R: Statistical Validation of Image Segmentation Quality Based on a Spatial Overlap Index. Acad Radiol 2004; 11:178–189[cited 2012 Oct 7]

9. Duncan KJ, Pattamadilok C, Knierim I, Devlin JT: Consistency and variability in functional localisers. Neuroimage 2009; 46:1018–1026

10. Corbetta M, Shulman GL: Control of goal-directed and stimulus-driven attention in the brain. Nat. Rev. Neurosci 2002; 3:201–215[cited 2010 Jan 3]

11. Wen X, Yao L, Liu Y, Ding M: Causal Interactions in Attention Networks Predict Behavioral Performance. J. Neurosci. 2012; 32:1284–1292[cited 2012 Sep 18]

12. Gilbert CD, Sigman M: Brain states: top-down influences in sensory processing. Neuron 2007; 54:677–696
